# Oncolytic herpes simplex viruses for the treatment of glioma and targeting glioblastoma stem-like cells

**DOI:** 10.3389/fcimb.2023.1206111

**Published:** 2023-05-31

**Authors:** Kimia Kardani, Judit Sanchez Gil, Samuel D. Rabkin

**Affiliations:** Department of Neurosurgery, Massachusetts General Hospital and Harvard Medical School, Boston, MA, United States

**Keywords:** glioblastoma, cancer stem cell, GSC, oHSV, oncolytic virus, immunotherapy, virotherapy

## Abstract

Glioblastoma (GBM) is one of the most lethal cancers, having a poor prognosis and a median survival of only about 15 months with standard treatment (surgery, radiation, and chemotherapy), which has not been significantly extended in decades. GBM demonstrates remarkable cellular heterogeneity, with glioblastoma stem-like cells (GSCs) at the apex. GSCs are a subpopulation of GBM cells that possess the ability to self-renew, differentiate, initiate tumor formation, and manipulate the tumor microenvironment (TME). GSCs are no longer considered a static population of cells with specific markers but are quite flexible phenotypically and in driving tumor heterogeneity and therapeutic resistance. In light of these features, they are a critical target for successful GBM therapy. Oncolytic viruses, in particular oncolytic herpes simplex viruses (oHSVs), have many attributes for therapy and are promising agents to target GSCs. oHSVs are genetically-engineered to selectively replicate in and kill cancer cells, including GSCs, but not normal cells. Moreover, oHSV can induce anti-tumor immune responses and synergize with other therapies, such as chemotherapy, DNA repair inhibitors, and immune checkpoint inhibitors, to potentiate treatment effects and reduce GSC populations that are partly responsible for chemo- and radio-resistance. Herein, we present an overview of GSCs, activity of different oHSVs, clinical trial results, and combination strategies to enhance efficacy, including therapeutic arming of oHSV. Throughout, the therapeutic focus will be on GSCs and studies specifically targeting these cells. Recent clinical trials and approval of oHSV G47Δ in Japan for patients with recurrent glioma demonstrate the efficacy and promise of oHSV therapy.

## Graphical Abstract

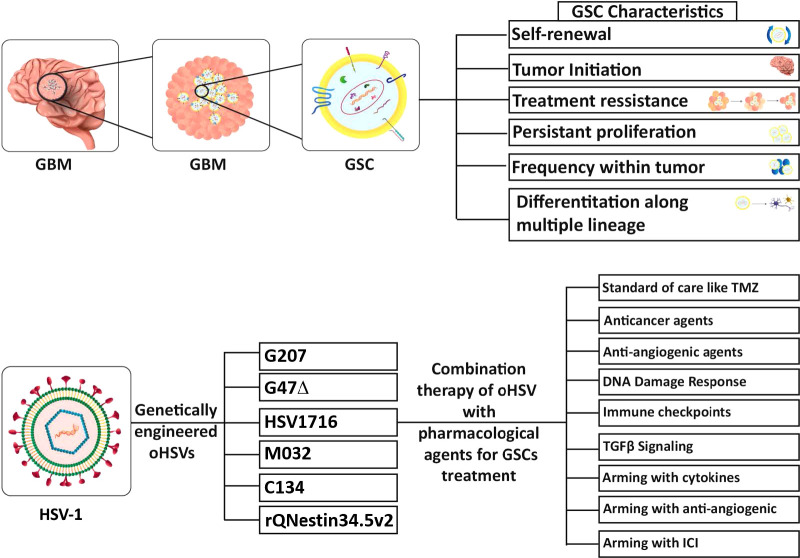

## 1 Introduction

Gliomas account for approximately 27% of primary, and 80% of all malignant central nervous system (CNS) tumors (Ostrom et al., 2020). Glioblastoma (GBM) comprises 54% of all gliomas and is the most malignant primary brain tumor in adults, currently classified as adult-type diffuse isocitrate dehydrogenase (IDH)-wildtype glioma, or grade 4 according to the fifth WHO (World Health Organization) classification of tumors of the central nervous system (WHO CNS5) (Louis et al., 2021). Unfortunately, GBM has a dismal prognosis and poor survival. Present standard-of-care treatment for primary GBM includes maximal safe surgical resection of the tumor, and radiotherapy with concomitant temozolomide (TMZ) chemotherapy (Stupp et al., 2009). After receiving standard treatment, patients’ overall median survival time is still only about 15 months with <10% of patients surviving over 5 years (Delgado-López and Corrales-García, 2016). This poor response to treatment leads to inevitable GBM recurrence within one year of primary diagnosis. This is due to the limitations of surgical resection given the infiltrating propensity of tumor cells, blood-brain-barrier, chemo- and radiotherapy resistance, immunosuppressive and pro-tumorigenic tumor microenvironment (TME), as well as extensive intratumoral heterogeneity and plasticity (Burster et al., 2021; Nguyen et al., 2021). Intratumoral and spatial heterogeneity arises from both the evolution of genomic alterations and variable gene expression profiles, which makes any targeted therapy unlikely to succeed (Sottoriva et al., 2013). While immunotherapy, in particular immune checkpoint inhibitors, has demonstrated exceptional outcomes in some patients in some cancers, the results in GBM have been disappointing, with three failed phase III clinical trials (Persico et al., 2021).

Oncolytic viruses (OVs) are a distinct class of cancer therapeutics, virotherapy, first clinically evaluated in the 1950’s, that exploits virus-host interactions and targeted viral proliferation (Saha et al., 2015; Zhang and Rabkin, 2021). They have two unique mechanisms of action: (i) selective replication in and killing of tumor cells while sparing normal cells and tissue, and amplifying *in situ* and spreading in the tumor; and (ii) exposing tumor antigens through immunogenic cell death and inducing inflammation, which promotes anti-tumor immunity (immunovirotherapy) (Zhang and Rabkin, 2021). This cancer selectivity is due to; a virus’s natural preference for replication in transformed cells (coxsackievirus, myxoma, Newcastle disease virus, parvovirus, reovirus, Seneca Valley virus), attenuation of vaccine strains (measles, vaccinia), and/or through genetic engineering (adenovirus, herpes simplex virus (HSV), poliovirus, vaccinia, vesicular stomatitis virus, zika) (Saha et al., 2015; Zhang and Rabkin, 2021). A wide range of OVs, including oncolytic HSV (oHSV), have been evaluated in clinical trials against various types of cancers, including GBM, which culminated in the recent approval of oHSV talimogene laherparepvec (T-Vec) for the treatment of advanced melanoma in the US and Europe (Zhang and Rabkin, 2021). OVs also provide a therapeutic platform that can deliver therapeutic genes for localized tumor expression, ‘armed’ OVs, such as T-Vec (Zhang and Rabkin, 2021). In this review, we will describe the genetic alterations endowing HSV with selectivity for GSCs, the current state of oHSV therapy for GBM, with a particular focus on targeting GSCs, and combinations with other therapeutic agents.

## 2 Glioblastoma stem-like cells

GBM stem-like cells (GSCs) are a major contributor to the features that make GBM such a difficult cancer to treat, and thus an important therapeutic target (Prager et al., 2020; Nguyen et al., 2021; Yabo et al., 2022). Cancer stem cells (CSCs) were first identified in leukemia, with the properties of proliferation, self-renewal, differentiation, and maintenance of the tumor (Lapidot et al., 1994). It was hypothesized that CSCs are a rare fraction of tumor cells with stem cell and tumor repopulating properties, that are at the apex of the tumor hierarchy (Kreso and Dick, 2014). Singh et al., were the first to isolate CSCs from human GBM specimens (GSCs, brain tumor-initiating cells (BTICs)), based on CD133 expression and culture in serum-free media with FGF and EGF (Singh et al., 2003). These cells had *in vitro* neural stem cell properties, such as sphere-formation and differentiation into more mature cellular lineages (Singh et al., 2003), and the ability to initiate tumor growth *in vivo* in immune-deficient mice (Singh et al., 2004). Human (h)GSCs are much more closely related to patients’ tumors than the classical glioma cell lines or primary serum-cultured GBM cells, based on transcriptomics and genomics (Lee et al., 2006). hGSC xenografts exhibit histopathological features of the patient’s tumor from which the GSCs were isolated (Singh et al., 2004; Wakimoto et al., 2012; Nigim et al., 2015). Multiple genomic features, such as somatic driver mutations, SNPs, and copy number alterations (CNAs), are conserved between GSCs and their parental tumors (Davis et al., 2016; Pesenti et al., 2019; Shen et al., 2019). Some alterations were gained or lost with GSC passage *in vitro* (Rosenberg et al., 2017) and gene expression and methylation patterns were more divergent (Shen et al., 2019). GSCs are reported to be chemo- and radio-resistant, due to upregulation of DNA damage response proteins, and enhanced survival *in vivo* (Bao et al., 2006; Ahmed et al., 2015). Conversely, variable chemotherapy sensitivity was seen with different hGSCs, some being sensitive and others resistant, generally representing the phenotype of the parental tumor from which they were isolated (Fouse et al., 2014) or the GSC cellular state (Segerman et al., 2016). For example, GSC sensitivity to TMZ correlated with O^6^-methylguanine-DNA methyltransferase (MGMT) methylation (Beier et al., 2012; Wakimoto et al., 2012), poly(ADP-ribose) polymerase (PARP) inhibitor with MYC expression (Ning et al., 2019), and epidermal growth factor receptor (EGFR) inhibitor with EGFR amplification (Tanaka et al., 2019). While a number of cell surface markers are enriched in GSCs (CD133, CD44, SSEA1/CD15, α6-integrin/CD49f, L1CAM, and A2B5) there are no definitive markers that can be used to identify GSCs in patient specimens, creating some controversy about their classification (Bhaduri et al., 2020; Prager et al., 2020; Suvà and Tirosh, 2020; Galdieri et al., 2021), so that GSCs described in different studies may actually reflect different cell populations.

GSCs can be differentiated *in vitro* by culture in serum, in the absence of growth factors, or with bone morphogenetic protein 4 (BMP4), where their morphology changes, they adhere to plastic, lose tumorigenicity and stem cell markers (CD133, nestin, Sox2, OLIG2), and gain lineage-specific markers (GFAP, MAP2, βIII-tubulin (TUJ1)) (Piccirillo et al., 2006; Wakimoto et al., 2009; Suva et al., 2014; Wang et al., 2018; Uneda et al., 2021). Differentiated GBM cells (DGCs) are also referred to as serum-cultured GBM cells (ScGCs) or bulk tumor cells. During the isolation of hGSCs from patient tumor specimens, GBM cells can also be cultured in serum to generate matched ScGCs and GSCs (Wakimoto et al., 2009). Differentiation occurs *in vivo* where implantation of GSCs generates tumors composed of differentiated tumor cells and a subpopulation of GSCs (Singh et al., 2004). In addition to neural lineages, GSCs can differentiate into endothelial cells and pericytes that incorporate into the tumor vasculature (Ricci-Vitiani et al., 2010; Wang et al., 2010; Cheng et al., 2013). DGCs have been shown to enhance GSC-derived tumor progression (Wang et al., 2018; Uneda et al., 2021). Epigenetic profiling of histone modifications at transcription factor loci and gene expression analysis revealed differences between GSCs, similar to neural stem cells, and DGCs (Suva et al., 2014). Based on this analysis, DGCs were reprogramed/dedifferentiated through the expression of 4 neurodevelopmental transcription factors (POU3F2, SOX2, SALL2, and OLIG2), in a similar fashion as fully differentiated normal cells reprogrammed into induced pluripotent stem cells (iPSCs) (Suva et al., 2014). This suggests an epigenetic plasticity to GSCs, which has been further delineated by single-cell gene expression studies (scRNA-seq) that define the phenotype of individual cells in a tumor and map the ‘putative cellular hierarchies’ (Suvà and Tirosh, 2020). Proliferative marker expression overlapped the stem cell signature, identifying cycling cells as GSCs (Suvà and Tirosh, 2020). The scRNA-seq data suggests 4 cellular states: neural progenitor cell (NPC)-like, oligodendrocyte progenitor cell (OPC)-like, astrocyte (AC)-like, and mesenchymal (MES)-like, with multiple states present in a single tumor (Neftel et al., 2019; Galdieri et al., 2021). All cellular states can efficiently propagate tumors in mice, with AC-like GSCs less effective (Suvà and Tirosh, 2020), and implantation of a single cell state propagates tumors with a mix of cell states, further illustrating the cellular plasticity and tumor heterogeneity in GBM (Neftel et al., 2019). Human GSCs and GSC-derived orthotopic xenografts provide representative GBM models that phenocopy the patients’ tumors to develop and test therapeutics for GBM, however, they can’t be grown in immunocompetent mice to study immune-mediated responses.

Mouse (m)GSCs provide important models for studying therapeutic approaches in immunocompetent mice, in particular immunovirotherapy, the immune-mediated effects of virotherapy (Cheema et al., 2013; Wouters et al., 2020; Rousso-Noori et al., 2021). The Verma group developed a lentivirus strategy to introduce Cre-lox-controlled activated oncogenes (i.e., Ras) and/or tumor suppressor gene (i.e., NF-1, p53) knock-downs in a specific cell type (i.e., GFAP+) in discrete regions of the mouse brain, from which mGSCs could be isolated (Marumoto et al., 2009). Tumor cells from the induced tumors fell into 3 of 4 cellular states (OPC-, AC-, and MES-like) identified in hGSCs, with multiple states seen in individual tumors, highlighting the similar plasticity between lentivirus-induced mouse and hGSCs (Neftel et al., 2019). One of these mGSCs, 005, was found to efficiently form non-immunogenic tumors in C57BL/6 mice that aren’t rejected and recapitulate many of the features of human GBM (Cheema et al., 2013). Among syngeneic murine GBM models, 005 mGSC-derived tumors most closely resembled patient GBM (Khalsa et al., 2020). Other GEM GBM tumor models used to isolate mGSCs include; (i) the RCAS/tv model developing classical, proneural, and mesenchymal GBM subtypes (Chen et al., 2020); (ii) GSCs isolated from sleeping beauty gene transfer induced GBM in mice (Calinescu et al., 2017); (iii) a GEM model where mGSCs, isolated from subventricular zone (SVZ)-driven tamoxifen-regulated Cre deletion of NF1, p53, and Pten induced tumors, were used to identify a quiescent gene signature that was conserved in hGSCs (Jin et al., 2021; Xie et al., 2022); and (iv) a GEM model (neural stem cell (NSC)-specific p53/Pten deletion) where mGSCs were serially passaged orthotopically in immunocompetent mice and the resultant brain tumors histopathologically resembled GBM, with a large infiltration of myeloid cells, and a cell-specific enrichment of subtype expression signatures (Costa et al., 2021). As immunotherapy becomes a larger component of cancer therapy, even though it is still lacking for GBM, the need for representative immunocompetent models of GBM is increasing. The established mouse glioma cell lines have problematic features for testing immunotherapy; they tend to be immunogenic, the tumors lack heterogeneity, and compared to patient tumors there are large differences in genomic alterations and expression profiles (Khalsa et al., 2020; Wouters et al., 2020; Jin et al., 2021). For the studies discussed below, we have used a rather broad description of GSCs, generally patient-derived primary GBM cells cultured in serum-free, growth factor-containing media, and/or described by authors as GSCs.

## 3 Making HSV oncolytic and safe in the brain

HSV belongs to the alphaherpesvirus subfamily of Herpesviridae, and contains an ~152 kb double-stranded DNA genome, encoding about 85 gene products, that is packaged in an enveloped icosahedral capsid (Peters and Rabkin, 2015). There are two human neurotropic alphaherpesviruses that have been used as OVs, HSV-1 and HSV-2 with very similar genomes, although HSV-1 is the predominant type (Peters and Rabkin, 2015). The virus lifecycle has three general phases; immediate-early (IE or α) involved in regulating gene expression, early (E or β) involved in DNA replication, and late (L or γ) involved in expression of structural proteins and virion assembly (Roizman and Zhou, 2015). HSV entry occurs in a multi-step process requiring viral glycoproteins gB, gD and gH/gL, with gD interacting with HSV receptors HVEM and Nectin-1. The binding sites for HVEM and nectin-1 are in the N-terminal 230 residues (Goins et al., 2016). Binding induces a conformational change in gD and the pro-fusion domain interacts with gH/gL heterodimer and/or trimer gH/gL/gB. Finally, gB is triggered to insert its fusion loops into the cell membrane leading to membrane fusion (Campadelli-Fiume et al., 2016). There are a number of properties of HSV that make it a particularly attractive OV: (i) many viral genes are non-essential for replication, including many required for pathogenicity, and can be deleted providing about 30 kb of space for insertion of therapeutic transgenes; (ii) it is a lytic virus that efficiently infects and proliferates, with a broad cellular and species tropism as wild-type; (iii) the virus genome is episomal and doesn’t integrate into host chromosomes minimizing insertional mutagenesis; and (iv) there are effective anti-viral drugs available (e.g. acyclovir) to treat unforeseen virus replication. Because of HSV pathogenicity in humans, especially when it enters the brain, engineering the virus for safety is of paramount importance.

**Figure 1:**
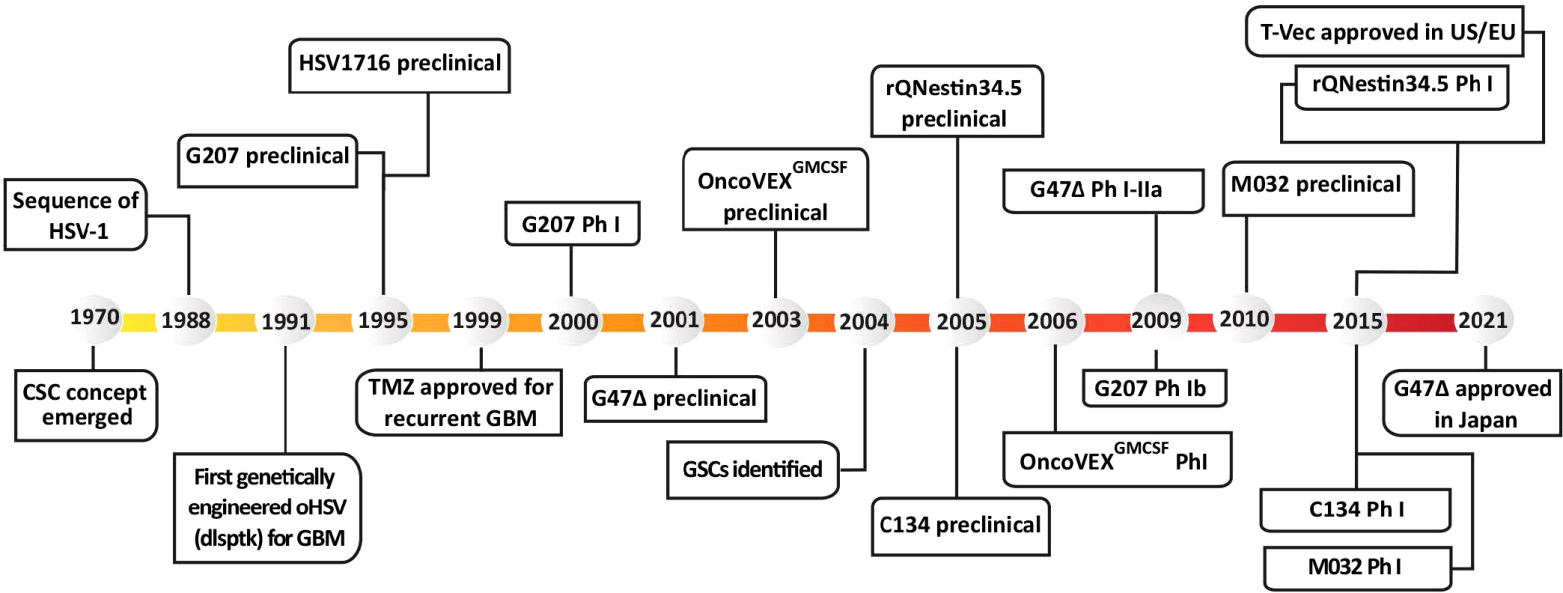
Historical timeline of oHSV and GSC development, from preclinical studies to clinical trials. T-Vec, previously known as Onco-VEX^GM-CSF^.

In 1991, the first genetically-engineered oHSV, *dl*sptk, for the treatment of glioma, with a thymidine kinase (TK) deletion, was reported (Martuza et al., 1991). However, the single TK deletion was not sufficiently attenuated for its use in the brain, and the lack of TK nullified nucleoside analog drug sensitivity, an important oHSV safety feature. This ignited a search for safer and more efficacious oHSVs that involved the following general strategies; (i) genetic alterations of other HSV genes that contribute to neuropathogenicity, nucleic acid metabolism, or apoptosis; (ii) retargeting virus entry; (iii) transcriptionally-targeted gene expression; (iv) ‘arming’ oHSV with therapeutic transgenes targeting tumor cells and/or ‘normal’ cells in the TME; and (v) combining oHSV with other pharmacological agents. GBM has been a favored disease target for oHSV, both preclinically and clinically with some success. Nonetheless, many of the oHSVs that have been developed are or are thought to be insufficiently safe in the brain, thus not discussed here. A historical timeline for the development of oHSV therapy for GBM is provided in **Figure 1**.

### 3.1 γ34.5 deletion mutants

**Figure 2:**
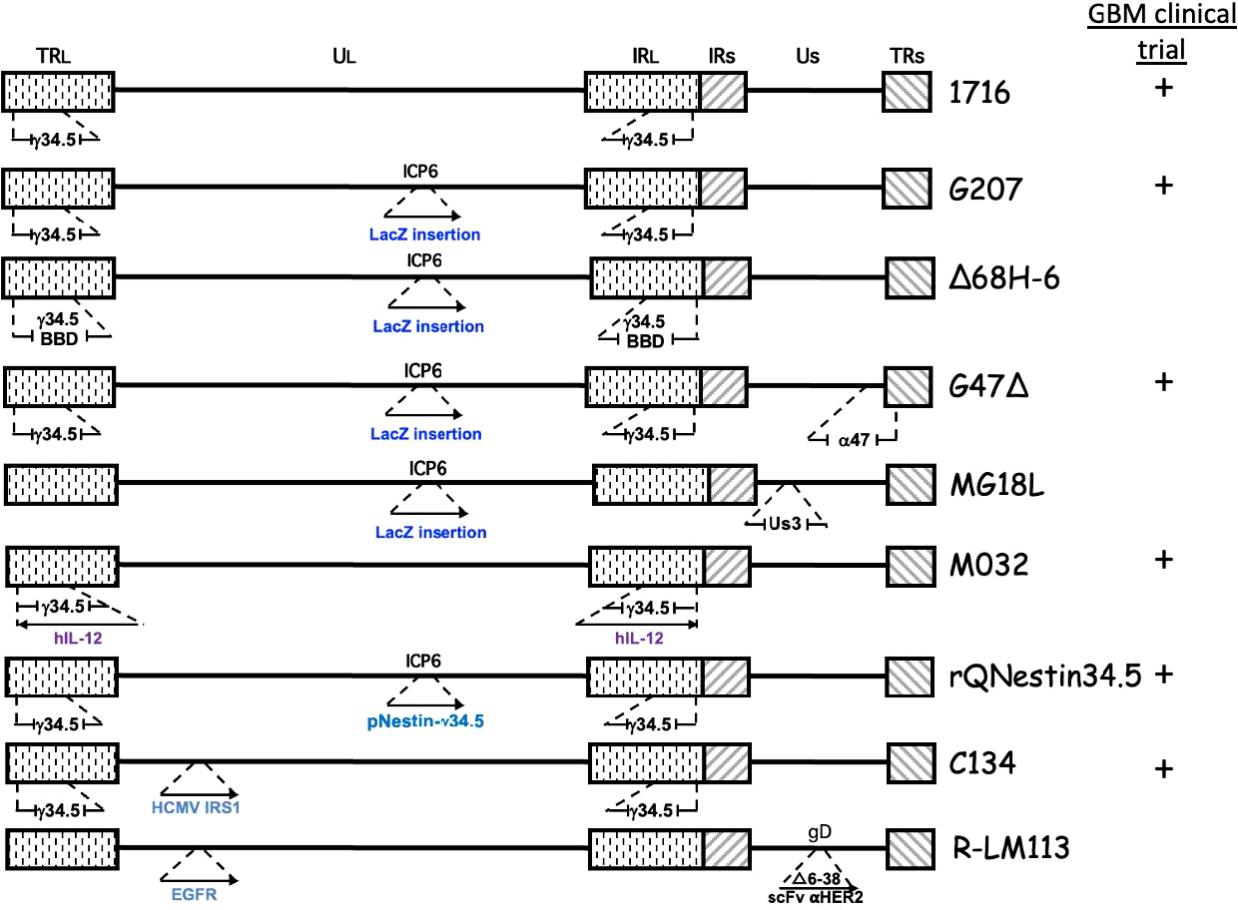
Genetic structure of oHSVs evaluated for GBM treatment. The genome consists of 2 unique regions (UL and US) flanked by terminal (TR) and internal (IR) repeats. *γ*34.5 is present in both Long repeats. Insertion of cDNA (→), beclin binding domain (BBD), deletion (
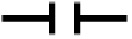
).

The diploid gene γ34.5 (RL1) encodes a multifunctional tegument protein that is the most common gene mutated or deleted in oHSVs targeting brain tumors, including all oHSVs in GBM clinical trials (**Table 1**; **Figure 2**). This is because γ34.5 is the major HSV gene driving neuropathogenicity (Peters and Rabkin, 2015). Among the other activities of γ34.5 are: (i) overcoming PKR-induced block to host protein shutoff, whereby its GADD34 homology domain promotes dephosphorylation of p-eIF2α and restoration of translation; (ii) blocking autophagy by binding beclin 1; and (iii) inhibition of RIG-I, TBK1, and cGAS/STING signaling that blocks IRF3 activation and type 1 interferon (IFN) signaling (Dogrammatzis et al., 2020; Kangas et al., 2021). Most tumors suppress antiviral innate immune responses (translation control and IFN responses) due to their anti-proliferative, pro-apoptotic, and immune activities (Du et al., 2017; Castiello et al., 2018; Boehmer et al., 2021; Musella et al., 2021; Solomon et al., 2022). Thus, γ34.5 deletions provide cancer selectivity, as well as significant safety in the brain. This was demonstrated with first-generation oHSVs, containing deletions of both copies of γ34.5 in different HSV parental strains, HSV1716 in strain 17 and R3616 in strain F, which were replication competent in human glioma and other cancer cell lines, and xenografts in immunodeficient mice (Jahan et al., 2021).

**Table 1:** oHSVs in clinical trials for Glioma.

oHSV Name	Genetic Alterations	Transgene	oHSV Ref	Disease	Current Developer	Clinical Trial Phase	Clinical Trial ID	Pre- or Clinical Ref
HSV1716 (Sephrevir^®^)	γ34.5Δ		(MacLean et al., 1991)	Recurrent HGG	Sorrento Therapeutics	1	NCT02031965	(Rampling et al., 2000; Papanastassiou et al., 2002; Harrow et al., 2004)
G207	γ34.5Δ, ICP6^-^	lacZ in ICP6	(Mineta et al., 1995)	Recurrent GBM Recurrent Ped HGG Ped Supratentorial Recurrent HGG Ped Cerebellar	Treovir, Inc	1/2	NCT00028158 NCT04482933 NCT02457845 NCT00157703 NCT03911388	(Markert et al., 2000; Markert et al., 2009; Markert et al., 2014; Friedman et al., 2021; Miller et al., 2022)
G47Δ (DELYTACT^®^; Teserpaturev)	γ34.5Δ, ICP6^-^, and ICP47Δ	LacZ in ICP6	(Todo et al., 2001)	Recurrent/Progressive GBM	Daiichi Sankyo, Inc.	1/2	UMIN000002661	(Todo et al., 2022a; Todo et al., 2022b)
C134 (MB-108)	γ34.5Δ	HCMV IRS1 driven by HCMV IEpro inserted in UL3-UL4 intragenic region	(Shah et al., 2007)	Recurrent malignant glioma	Mustang Bio	1	NCT03657576	(Cassady et al., 2017)
rQNestin34.5v.2 (CAN-3110)	γ34.5Δ, ICP6^-^	γ34.5 driven by the nestin enhancer-hsp68pro in ICP6	(Kambara et al., 2005)	Recurrent malignant glioma	Candel Therapeutics	1	NCT03152318	(Chiocca et al., 2020)
M032 (NSC-733972)	γ34.5Δ	IL-12 driven by murine early-growth response-1 pro (Egr-1) in γ34.5	(Roth et al., 2014)	Recurrent or new GBM	University of Alabama at Birmingham	1/2	NCT02062827 NCT05084430	(Patel et al., 2016)

Ped, pediatric; Pro, Promoter; Ref, References.

HSV1716 (Seprehvir; Sorrento Therapeutics) was the first oHSV to enter clinical trial in Europe, with 3 trials in GBM patients (**Table 1**). The first study enrolled 9 patients with recurrent high-grade gliomas (HGG) who received escalating intratumoral doses of 10^3^-10^5^ pfu in 1 ml. Four of nine patients survived longer than 14 months, and no evidence of virus shedding or reactivation was detected, demonstrating the safety and feasibility of HSV1716 (Rampling et al., 2000). In the second study, HGG patients had their tumors resected 4-9 days after intratumoral treatment to evaluate virus replication. Infectious virus, more than input, was recovered at the injection site in two of twelve patients, both being seronegative and seroconverted, while virus DNA was detected in 10 patients with 4 being at distal sites (Papanastassiou et al., 2002). Finally, in a third study, virus (10^5^ pfu in 1 ml) was injected into the rim of the resection cavity after maximal surgical resection, with 3 of 12 patients alive and stable after 15-22 months (Harrow et al., 2004). None of the patients in any of the 3 clinical trials experienced adverse events attributable to the virus (Harrow et al., 2004). There was concern about the safety of oHSVs with only a single genetic alteration, even deletion of γ34.5, which is a reason why the HSV1716 clinical trials dose escalated only up to 10^5^ pfu. This prompted the development of multimutated second-generation oHSVs, of which G207 is the exemplar.

### 3.2 Nucleotide metabolism gene mutations

HSV encodes a number of proteins involved in nucleotide metabolism in order to replicate in non-cycling post-mitotic cells. Mutations in these genes confer specificity for dividing cells (ie., cancer cells), which express cellular nucleotide metabolism enzymes, and often also attenuate pathogenicity, making them important mutations for oHSV. In addition to TK, mutations in uracil DNA glycosylase (UNG or UL2) and ICP6 (UL39) have been used to construct oHSVs (Pyles et al., 1997; Peters and Rabkin, 2015). ICP6 is the large subunit of the viral ribonucleotide reductase (RR), an enzyme that converts ribonucleotides (ribonucleoside diphosphate, NDPs) into deoxyribonucleotides (dNDPs). RR activity is encoded in the C-terminus and is required for viral DNA replication (Peters and Rabkin, 2015). The N-terminal RHIM domain binds RIP3 to trigger necroptosis (Wang et al., 2014). In contrast to TK mutants, ICP6 and UNG mutants are hypersensitive to nucleoside analog drugs (Mineta et al., 1994; Pyles et al., 1997). ICP6 mutants are attenuated in pathogenicity, including in the brain. An ICP6 mutated oHSV, hrR3, with an *E. coli* LacZ insertion inactivating ICP6, and forming a fusion protein, was efficacious in inhibiting the growth of orthotopic glioma xenografts and spreading in the tumors (staining for LacZ) (Mineta et al., 1994). LacZ provides a reporter gene, to easily evaluate virus spread, and as a unique identifier for oHSV, enabling discrimination with patient clinical isolates during clinical trials. Because of these properties, ICP6 mutations have been combined with other mutations to enhance oHSV safety, especially in the brain, and glioma specificity; including, 1716-6 (γ34.5Δ ICP6^-^) from HSV1716 (γ34.5Δ), MG18L (Us3^-^ ICP6^-^) from R7041 (Us3^-^), and Δ68H-6 (γ34.5-BBDΔ ICP6^-^) from Δ68H (γ34.5-BBDΔ) (Peters and Rabkin, 2015).

### 3.3 γ34.5Δ - ICP6 multimutant

G207 (**Table 1**; **Figure 2**) was constructed with the goal of clinical translation to patients with GBM. Thus, safety was of paramount importance. It was derived from R3616 (γ34.5 deleted (Δ)) with an inactivating insertion of LacZ into the ICP6 gene, as in hrR3 (Mineta et al., 1995). From a safety point of view, it has a number of important elements: (i) two pathogenicity genes mutated/deleted that are broadly spaced in the genome making recombination/reversion highly unlikely; (ii) hypersensitivity to anti-viral nucleoside analog drugs; (iii) temperature-sensitivity to compromise replication under fever conditions; (iv) selectively grows in and kills glioma cells and not astrocytes or neurons; and (v) is safe after intracerebral injection in mice and non-human primates, as opposed to wild-type HSV at a 10^4^ lower dose (Mineta et al., 1995). In addition, preclinical studies in immunocompetent mouse models provided the first demonstration of OV-induced *in situ* cancer vaccination (Toda et al., 1999), including in mice with both subcutaneous and intracerebral tumors, where G207 treatment of the subcutaneous tumor inhibited the growth of the intracerebral tumor and provided protection to tumor rechallenge in the brain (Todo et al., 1999; Jahan et al., 2021).

G207 was the first oHSV to enter clinical trial in the US in a dose-escalation (10^6^ - 3 x 10^9^ pfu) phase I study for recurrent HGG (Markert et al., 2000). G207 was administered stereotactically to a single site in a contrast-enhancing region, except for the final dose. There were no dose-limiting toxicities attributable to G207, and responses were not dose-dependent and seen at low doses. For example, the longest surviving GBM patient (>17 months from treatment) received a dose of 3 x 10^7^ pfu (Markert et al., 2000). In a follow-on phase Ib trial, G207 was inoculated *via* intratumoral catheter (1.5 x 10^8^ pfu) followed by tumor resection 2-5 days later and virus injection (1 x 10^9^ pfu) adjacent to the resection cavity (Markert et al., 2009). Four of six patients displayed increased CD3+ cells (IHC) post-G207, and viral DNA was detected in all patients’ tumors by PCR. Two patients had HSV-1 detected in their saliva at 7 days post-resection, however, these were demonstrated to be wild-type virus based on negative PCR for LacZ, while no spread of G207 was detected in serum, conjunctival swabs, or saliva (Markert et al., 2009). The final clinical trial with G207 for adult GBM was in combination with radiation (5 Gy) 24 hr post-G207 (Markert et al., 2014). In this trial, 5 of 8 patients were seronegative, much lower than in the 2 previous trials at 32%, and only 1 seroconverted (Markert et al., 2014). Like the two prior trials, treatment was well tolerated, and no patients developed encephalitis or serious adverse events attributed to the virus, G207 was not detected in the saliva or serum, and some patients had significant responses (Markert et al., 2014).

A recently completed phase I clinical trial of G207 in pediatric HGG has reported promising results, including a median OS of 12.2 versus 5.6 months in historical controls (Friedman et al., 2021). All 12 patients were IDH1 wild-type without favorable H3.3 mutations. The trial included 4 groups, a single administration of 10^7^ or 10^8^ pfu *via* slow infusion from 1-4 catheters with or without 5 Gy of radiation. G207 was not associated with any grade 2-4 adverse events or evidence of peripheral shedding (saliva). One patient with a saliva-positive HSV PCR result was LacZ negative demonstrating reactivation of a clinical strain (Friedman et al., 2021). Seroconversion occurred in 3 of 9 seronegative patients, all at the 10^8^ pfu dose. Intriguingly, the median OS was 5.1 months for 3 seropositive patients and 18.3 months for those that seroconverted. Significant increases in tumor-infiltrating lymphocytes, both CD4+ and CD8+ cells, were detected in patient biopsies 2-9 months post-treatment, while no HSV-1 staining was observed (Friedman et al., 2021).

While the γ34.5 deletion has a large impact on attenuating pathogenicity and replication in normal cells, it also attenuates virus replication and cell killing in cancer cell lines (Todo et al., 2001; Shah et al., 2007; Kanai et al., 2012b). Once human GSCs were identified and isolated, it was possible to ask whether they were permissive to γ34.5Δ oHSV replication. Unfortunately, γ34.5Δ oHSVs (1716, R3616, G207) replicate poorly, if at all, in patient-derived GSCs *in vitro* that have been tested (Wakimoto et al., 2009; Kanai et al., 2012b; Peters et al., 2018). However, they do replicate well in patient-matched serum-cultured GBM cells (ScGCs), similar to glioma cell lines, which were used in the preclinical studies (Peters et al., 2018). This is due to a block in true late viral gene translation in GSCs (Peters et al., 2018). Virus yield of γ34.5Δ C101 (parent of C134 (**Figure 2**)) in 3 human patient-derived xenograft (PDX)-derived GSC xenolines was 10^2^ - 10^4^-fold lower than with wild-type HSV, and somewhat decreased or not in hypoxia where CD133+ cells were greatly increased (Friedman et al., 2012). In a comparison of stem-like cells from patient-derived pediatric high-grade brain tumors with adult GBM, pediatric brain tumor xenograft stem-like cells were ≥ 6-fold more sensitive to G207 cytotoxicity than cells from adult tumors, having a mean sensitivity at MOI~3 after 3 days of only 46% (Friedman et al., 2018). Pediatric tumor GSCs expressed significantly higher levels of HSV entry mediator nectin-1 (CD111) than adult GSCs, which inversely correlated with IC50 (Friedman et al., 2018). In one pediatric embryonal supratentorial primitive neuroectodermal tumor (sPNET) stem-like cell-derived intracranial xenograft model, G207 significantly extended survival, whereas, in 3 different GSC-derived intracranial tumor models, irrespective of nectin-1 expression, G207 extended survival, but not significantly (Friedman et al., 2018).

### 3.4 ICP47 complementation of γ34.5Δ

This limitation of γ34.5Δ oHSVs led to strategies to overcome the attenuated virus replication without significantly increasing pathogenicity, including second-site suppressors (G47Δ), targeted mutations in γ34.5 (Δ68H-6), and transcriptionally-targeted γ34.5 expression (rQNestin34.5). Initially, an *in vitro* screen for second-site suppressor mutations of γ34.5Δ virus replication in non-permissive cancer cells was performed. This identified deletions of ICP47 (Us12, α47) and the Us11 late promoter (Mohr and Gluzman, 1996). This deletion placed the late Us11 gene under control of the Us12 IE promoter, allowing US11 to be expressed as an early instead of late protein (Mulvey et al., 1999). Us11 binds to double-stranded RNA, blocking PKR activation and eIF2α phosphorylation, thus complementing the lack of γ34.5 (Peters and Rabkin, 2015). ICP47 binds to transporter associated with antigen processing (TAP) to prevent MHC I expression, shielding virus-infected cells from CD8+ T cells (Todo et al., 2001; Dogrammatzis et al., 2020). Unfortunately, ICP47 activity is species-specific and minimally active in rodents (Ahn et al., 1996), so in mice, HSV-1 doesn’t inhibit TAP, and ICP47Δ oHSV behaves like ICP47^+^ in humans making it difficult to study the effects of ICP47Δ. This could be somewhat circumvented by using ‘humanized’ mice, immunodeficient mice with human immune cells.

G47Δ (Delytact®, Teserpaturev; Daiichi Sankyo) (**Table 1** and **Figure 2**) is a third-generation oHSV engineered from G207 by deletion of ICP47 and the Us11 promoter (Todo et al., 2001). It replicates in and kills glioma cells, and importantly GSCs, and inhibits tumor growth more effectively than G207 (Wakimoto et al., 2009). Intracerebral injection of G47Δ in A/J mice was as safe as with G207 (Todo et al., 2001). There have been two clinical trials of G47Δ for recurrent HGG in Japan. A phase I/II clinical trial of 13 patients with progressive or recurrent GBM at the University of Tokyo where 3x10^8^ pfu (3 patients) or 1x10^9^ pfu was injected two times (5-14 days apart) at the same coordinates (UMIN000002661) (Todo et al., 2022a). The median overall survival was 30.5 months from initial diagnosis and the 1-year survival rate from the last G47Δ administration was 38.5%, with 3 patients surviving more than 46 months; none were IDH1 mutants (Todo et al., 2022a). On MRI, all patients typically showed clearing at the injection site and enhancing lesion enlargement (immunoprogression) after the first G47Δ injection. HSV staining was negative at autopsy, while an influx of CD4+ and CD8+ cells were seen in biopsies at the second virus injection and persisted at autopsy (Todo et al., 2022a). Seroconversion occurred one week after G47Δ in all four seronegative patients. The most common G47Δ-related adverse events were headache, fever, or vomiting in 12 of the patients, with no evidence of virus shedding (blood, urine, saliva) within two weeks of treatment (Todo et al., 2022a). This first study demonstrated the safety and potential therapeutic activity of G47Δ for GBM.

In the registration single-arm phase II trial with 19 patients with recurrent GBM, G47Δ (1x10^9^ pfu in 1 ml) was stereotactically injected into 1-3 sites up to 6 times, at intervals of 5-14 days for doses 1 and 2, and 2-6 weeks for the remainder (Todo et al., 2022b). The primary endpoint of 1-year survival was met by 84% of patients, leading to a conditional approval of G47Δ for the treatment of malignant glioma in Japan, the first OV approved in Japan (Daiichi-Sankyo, 2021). Median overall survival was 28.8 months from the first surgery/diagnosis, with 3 patients alive over 3 years from the last viral dose. Interestingly, the median OS was virtually the same in the 6 IDH1 mutant patients as in the IDH1 wildtype patients (Todo et al., 2022b). These results were better than the 20.9 months seen in the randomized phase III clinical trial of tumor-treating fields and TMZ maintenance in patients who had completed standard therapy (Stupp et al., 2017). The magnetic resonance imaging (MRI) observations were informative and consistent with other oHSV trials for glioma; contrast-enhancement clearing at the injection site, some enlargement of target lesions right after virus injection (immunoprogression), and reductions in tumor size that often took over 9 months (Todo et al., 2022b). An important feature of this trial was the acquisition of tumor biopsies before each virus injection, which were used to evaluate histology and T cell infiltration. CD4+ T cells tended to infiltrate at the 2^nd^ injection, a little sooner than CD8+, but both increased with later injections and persisted after treatment ceased for up to 50 months, while the number of Foxp3+ T regulatory cells (Tregs) was very low throughout (Todo et al., 2022b). Despite this, tumor shrinkage took 4 months or more to detect. The safety profile was good, with only 2 grade 3 events (fever and vomiting) attributable to the virus and frequent low-grade fever and headache. A survival benefit without remarkable tumor responses (ORR of only 5.3%), delayed time to treatment response by MRI after an initial inflammatory response, and an early influx of T cells may be characteristics of oHSV therapy in GBM.

### 3.5 Overcoming γ34.5Δ attenuation

Δ68H-6 (**Figure 2**; **Table 2**) has a deletion in the γ34.5 beclin 1 binding domain (BBD; 68 to 87 aa) and LacZ inactivated ICP6 (Kanai et al., 2012b). Amongst other activities, γ34.5 inhibits autophagy by binding to beclin 1 through the BBD, which modestly reduces neurovirulence (Talloczy et al., 2006; Orvedahl et al., 2007). Δ68H-6 was not as effective at inhibiting p-eIF2α as its BBD rescued virus (Δ68HR-6), but better than γ34.5Δ oHSV (Kanai et al., 2012b). However, it replicated in and killed GSCs as well as γ34.5+ oHSVs, and inhibited the growth of GSC-derived orthotopic tumors, while causing only transient and minimal neurologic symptoms (Kanai et al., 2012b).

**Table 2:** oHSVs targeting GSCs. Genome structure, activity, and GSC models.

Name	Genetic alterations	GSC Ref	In vivo model	Main results	Ref
RAMBO	-γ34.5Δ -Armed with Vasculostatin (Vstat120), under IE4/5pro	(Wakimoto et al., 2012)	MGG23 implanted in immuno-deficient mice	-RAMBO extended median survival. -Vstat120 synergized with bevacuzumab and reduced migration and invasion.	(Tomita et al., 2019)
VAE	-γ34.5Δ, ICP6^-^, -Armed with endostatin-angiostatin fusion	(Zhang et al., 2014)	GBM-SC implanted in nude micee	-VAE extended median survival over parental virus or recombinant endtostatin. -Decreased microvessel density.	(Zhang et al., 2014)
MG18L	ICP6^-^, LacZ^+^, Us3Δ	(Wakimoto et al., 2009; Wakimoto et al., 2012)	BT74, MGG4 or MGG31 implanted in athymic mice	-PI3K/Akt inhibitor + MG18L kills GSCs not astrocytes. -PI3K/Akt inhibitor + MG18L prolongs survival to 50% cures. -Combination of PARP*i* + MG18L kills PARP*i*-sensitive and -resistant GSCs *in vitro* and extends median survival. -Combination of TGF-β inhibitors + MG18L increase recurrent GSC killing. -TGF-β inhibitors + MG18L result in 60% cured mice.	(Kanai et al., 2011; Esaki et al., 2017; Ning et al., 2017)
Δ68H-6	γ34.5 BBD deleted, ICP6^−^, LacZ^+^	(Wakimoto et al., 2009)	MGG4 implanted in athymic mice	-Δ68H-6 increased survival. -Δ68H-6 is safe.	(Kanai et al., 2012b)
NG34	-γ34.5Δ, ICP6^−^. -hGADD34 driven by the nestin enhancer-hsp68pro	(Nakashima et al., 2018)	G35 implanted in athymic mice	-more cytotoxic in hGSCs than rQNestin34.5, but less neurotoxic. -extended survival of hGSC-bearing mice.	(Nakashima et al., 2018)
G47Δ-mIL12	-γ34.5Δ, ICP6^−^, ICP47Δ, LacZ^+^. -Armed with mIL12 under HCMVpro.	(Cheema et al., 2013)	005 implanted in C57/BL6 mice	-antiangiogenic activity in vitro and in vivo. -Extends survival of mice with 005 tumors, dependent on T cells.	(Cheema et al., 2013)
G47Δ-mAngio	-γ34.5Δ, ICP6^−^, LacZ^+^, ICP47/Us11proΔ, -Armed with murine angiostatin (mAngio)	(Wakimoto et al., 2009)	MGG4 implanted in athymic mice	-G47Δ-mAngio prolonged mouse survival. -G47Δ-mAngio combined with G47Δ-mIL12 increased survival, virus spread, and decreased macrophages.	(Zhang et al., 2013)
OV-Cmab-CCL5	-γ34.5Δ, ICP6^−,^GFP^+^ -Armed with Cetuximab-CCL5 fusion protein under IE4/5pro.	(Uchida et al., 2013)	GBM30-FFL implanted in NSG mice	-Multi-mechanistic efficacy in immuno-deficient and -competent -Increased migration of NK, macrophages, CD4^+^ and CD8^+^T cells. -OV-Cmab-CCL5 improves median survival 2.4-fold.	(Tian et al., 2022)
OV-αCD47-G1	-γ34.5Δ, ICP6^−^, GFP^+^ -Armed with αCD47-IgG1 under IE4/5pro.	(Xu et al., 2021)	GBM43 implanted in athymic mice	OV-αCD47-G1 treatment *in vivo* releases αCD47 into the TME and prolongs survival.	(Xu et al., 2021)
OV-IL15C	-γ34.5Δ, ICP6^−^, GFP^+^ -Armed with hIL15-IL15Rα sushi domain under IE4/5pro.	(Uchida et al., 2013)	GBM30 implanted into NSG mice	-hGSC killing by NK cells treated with conditioned media. -extended survival with hCD8+ T cells and with EGFR-CAR NK.	(Ma et al., 2021)
oHSV-TRAIL	-γ34.5Δ, ICP6^−^, LacZ^+^, ICP47/Us11proΔ -Armed with TRAIL under IE4/5pro.	(Wakimoto et al., 2012; Esaki et al., 2017)	MGG23 and MGG31 implanted in athymic mice	oHSV-TRAIL increased survival in mice with TMZ-resistant primary and recurrent GSCs	(Jahan et al., 2017)
OV-CDH1	-γ34.5Δ, ICP6^−^, GFP^+^ -Armed with e-cadherin (CDH1) under IE4/5pro.	(Uchida et al., 2013)	GBM30 implanted in athymic mice	OV-CDH1 mediated enhanced viral spread and increased NK infiltration.	(Xu et al., 2018)
KNE	-gB:NT, scFv EGFR-retargeted gD Δ224-38.	(Uchida et al., 2013)	GBM30 implanted in athymic mice	-Antitumor efficacy in vivo, >50% long-term survivors. -safe in the brain of nude mice.	(Uchida et al., 2013)
KGE4:T124	-γ34.5Δ, ICP6^−^ -4x miR-124 target sequence in 3’UTR of ICP4 -EGFR-retargeted gD.	(Uchida et al., 2013)	GBM30 implanted in BALB/c athymic mice	miR-124T sites in ICP4 gene did not affect antitumor efficacy	(Mazzacurati et al., 2015)
R-LM113	-scFv HER2-retarged gD -wild-type backbone	(Calzolari et al., 2008)	-mGBM-HER2 implanted in C57/BL6 mice. -BALB/c-HGG-HER2 in BALB/c mice.	Antitumor efficacy in immunocompetent mouse models.	(Gambini et al., 2012; Reisoli et al., 2012)
R-613	-scFv EGFRvIII-retargeted gD Δ6-38 -wild-type backbone.	(Mazzoleni et al., 2010)	L0306 implanted in NOD/SCID mice	-R-613 infects EGFRvIII^+^ GSCs and spreads *in vitro and in vivo*. -Early, but not late treatments increase mice survival.	(Appolloni et al., 2021)
R-115	-scFv HER2-retarged gD. -wild-type backbone. -Armed with mIL12 under HCMVpro.	(Calzolari et al., 2008)	-mHGG^pdgf^-hHER2 implanted in C57/BL6 mice.	-Significant improvement of overall median survival, but not different than R-LM113. -30% of mice cured.	(Alessandrini et al., 2019)
oHSV/Nb-gD	-γ34.5Δ, ICP6^−^, GFP^+^, ICP47Δ. -Nanobody-hCXCR4 retargeted gD.	(Sanchez Gil et al., 2022)	T033 implanted in athymic mice	-Infected CXCR4-expressing GSCs. -Treatment of mice with T033 tumors did not extend survival.	(Sanchez Gil et al., 2022)

BBD, Beclin-1 binding domain; CM, conditioned media; MMP9, Matrix Metalloproteinase 9; N/A, not applicable; NSG, NOD/SCID/IL2rg; PARPi, Poly-ADP-ribose polymerase inhibitor; ULBP3, UL16 binding protein 3; VEGF, vascular endothelial growth factor.

Alternate strategies to overcome γ34.5Δ attenuation include expressing a mammalian or viral orthologue of γ34.5. C134 (**Figure 2**; **Table 1**) has the HCMV IRS1 gene, which prevents PKR activation, under control of the HCMV IE promoter inserted into the UL3-UL4 intragenic region of γ34.5Δ R3616 (Cassady, 2005). C134 infection of glioma cell lines restores late viral protein synthesis and replication close to levels of wild-type HSV-1 (Shah et al., 2007), such that C134 replicated much better in GSCs than C101 (γ34.5Δ) *in vitro* (Friedman et al., 2015). At a low dose, C134 was significantly better at extending the survival of mice with intracranial glioma cell line xenografts than C101, which was not significantly better than saline (Shah et al., 2007). C134 was found to be safe after intracerebral injections of HSV-1 susceptible mice and non-human primates (1 x 10^7^ pfu) (Cassady et al., 2017), and a phase I clinical trial for recurrent GBM patients is now recruiting (NCT03657576).

rQnestin34.5v2 (**Figure 2**; **Table 1**) has a copy of γ34.5 under the control of the nestin promoter inserted into the ICP6 locus of a γ34.5Δ virus (Kambara et al., 2005). Nestin is upregulated in neural progenitor cells and glioma and is a stem cell marker for GSCs (Ludwig and Kornblum, 2017; Xie et al., 2021). rQnestin34.5v2 replicated better and was more cytotoxic than parental rHsvQ1 (γ34.5Δ) in glioma cell lines and GSCs while retaining non-permissivity in normal cells (Kambara et al., 2005; Chiocca et al., 2020). *In vivo*, rQnestin34.5v2 treatment doubled the survival of athymic mice bearing U87 glioma tumors compared to the control virus (rHsvQ1) (Kambara et al., 2005). Moreover, rQnestin34.5v2 was not toxic, with no adverse effects detected in mice (Chiocca et al., 2020). A phase I clinical trial was approved in 2017 to test the safety of the virus in humans and to define the correct dose of virus to be administrated (NCT03152318). NG34 is similar to rQnestin34.5, except the nestin promoter drives expression of human GADD34, which is homologous to the carboxy-terminal PP1 domain of γ34.5 (Nakashima et al., 2018) (**Table 2**). It is as cytotoxic as rQnestin34.5 *in vitro* to GSCs and *in vivo* at inhibiting GSC-derived tumor growth, and more cytotoxic than parental rHsvQ1 (Nakashima et al., 2018). Interestingly, it is less pathogenic than rQnestin34.5 after intracerebral injection in BALB/c and athymic mice, suggesting γ34.5 has additional pathogenicity activities (Nakashima et al., 2018).

Another way to overcome γ34.5Δ oHSV attenuated replication in cancer cells and especially its inability in GSCs, is to introduce mutations in other genes endowing oncolytic activity in the presence of γ34.5. MG18L (**Figure 2**; **Table 2**) contains a deletion of the US3 gene, as well as the LacZ inactivation of ICP6 (Kanai et al., 2011). U_S_3 is a non-essential gene encoding a serine-threonine kinase that blocks apoptosis and Akt activation (Leopardi et al., 1997). Apoptosis-related pathways are altered/dysfunctional in 80% of GBMs (Parsons et al., 2008). Thus, MG18L provides tumor specificity by enhancing apoptosis in normal cells, blocking virus replication and spread, and also to some extent in GSCs (Kanai et al., 2011). The ED50 of MG18L is about 4-fold lower in GSCs, similar to G47Δ, than in astrocytes. In addition, MG18L induces PI3K/AKT signaling, so that it synergizes with PI3K inhibitors in most GSCs, an interaction not induced by G47Δ (Kanai et al., 2011). Intracerebral injection of 4 x 10^6^ pfu of MG18L caused only minor and transient neurologic deficits in 25% of mice, illustrating its safety, similar to G47Δ (Kanai et al., 2011). In a GSC-derived xenograft model, a single intratumoral injection of MG18L significantly extended survival by 25% and increased apoptosis in the tumor, while the addition of a PI3K/AKT inhibitor resulted in 50% long-term survivors (Kanai et al., 2011). MG18L also exhibits either synergy or additivity with TGFβ receptor inhibitor and PARP inhibitors in GSCs *in vitro* and *in vivo* (Esaki et al., 2017; Ning et al., 2017).

### 3.6 Retargeted oHSV

An alternate strategy to endow HSV with oncolytic activity and patient safety is to retarget oHSV to only infect specific cancer cell types by detargeting its natural receptors and targeting cancer-specific cell surface molecules (Campadelli-Fiume et al., 2016; Goins et al., 2016). If this targeting is specific enough and limited to cancer cells, there should be no need to mutate virus genes for cancer selectivity and safety. HSV entry occurs in a multiple step process in which essential glycoproteins gB, gD and gH/gL are required, with gD interacting with HSV receptors HVEM and Nectin-1 (Goins et al., 2016). Therefore, most oHSV retargeting strategies are based on modifying gD, however, ligand insertions into gB and gH have also been reported (Campadelli-Fiume et al., 2016; Goins et al., 2016). Nectin-1 expression (CD111; HSV receptor) ranged from 4-76% of adult GBMs and inversely correlated with the IC50s of G207 in adult GBM, including PDX-derived GSCs, and pediatric brain tumors (Friedman et al., 2018). This provides a rationale for targeting other non-HSV receptor cell surface molecules in GBM. Conversely, it also illustrates a major downside to targeting specific cell surface molecules; the inherent heterogeneity of their expression.

Epidermal growth factor receptor (EGFR) is overexpressed in 60% of primary GBMs (Hersh et al., 2022). KNE (**Table 2**) was retargeted to human EGFR- and EGFRvIII-overexpressing cells by inserting an scFv against EGFR into gD, which was also mutated for the HVEM and nectin-1 binding sites, and with gB mutations that enhance entry (Uchida et al., 2013). KNE infection was selective for EGFR-overexpressing cells *in vitro* and inhibited GSC-derived tumor growth in immunodeficient mice (Uchida et al., 2013). R-LM113 (**Figure 2**; **Table 2**) targets human HER2 (EGFR2, erbB2) (Menotti et al., 2008), which is expressed in a majority of GBMs, as well as GSCs (Ahmed et al., 2010). It efficiently infected hGSCs and was efficacious in orthotopic glioma implant models with hHER2+ murine glioma cells in immunodeficient and immunocompetent mouse models (Gambini et al., 2012; Alessandrini et al., 2019). R-613, similar to R-LM113 except with an scFv to EGFRvIII inserted into gD, effectively infected hGSCs expressing EGFRvIII but not those without (Appolloni et al., 2021) (**Table 2**). CXCR4+ cells were targeted using a nanobody sequence against human CXCR4 inserted into gD (oHSV/Nb-gD), as in KNE (Sanchez Gil et al., 2022). oHSV/Nb-gD was constructed on a G47Δ-like parent (γ34.5Δ, ICP6^-^, and ICP47Δ) (**Table 2**). CXCR4 is overexpressed in GBM and GSCs, and correlates with tumor size, progression, and recurrence, while the CXCL12/CXCR4 pathway is associated with GBM cell migration. oHSV/Nb-gD demonstrated efficacy in orthotopic xenografts of GSCs and U87MG.CXCR4+ cells (Sanchez Gil et al., 2022). Unfortunately, scFv’s and nanobodies against human cell surface molecules are usually species-specific, so proper toxicity testing of retargeted viruses for off-cancer target effects on normal cells cannot be evaluated in mice.

Another means to decrease pathogenicity and replication selectively in normal cells is the use of miR target sequences to repress virus gene expression in normal cells that express that miR. KG4:T124 is KG with an insertion of target sequences to miR-124, which is expressed in healthy neurons but not in GBM cells (Gaur et al., 2007), into the 3’UTR of ICP4 to block virus replication in the brain, but not in GSCs, and is also deleted for the IR/joint region and ICP47, and contains mutant gB (Mazzacurati et al., 2015). Other miRs that are selective for the brain include miR-128, -137, -219a, and -204, which have been incorporated into ONCR-159 (Kennedy et al., 2020). KGE4:T124 is KG4:T124 retargeted to human EGFR, as in KNE, so that it only infects human EGFR-overexpressing cells, and its replication is restricted to cells that do not express miR-124 (Mazzacurati et al., 2015) (**Table 2**). It replicated as well as parental KG in hGSCs *in vitro* and inhibited GSC-derived tumor growth *in vivo*. In contrast to KG, KG4:T124 was non-toxic after intracranial injection in mice (Mazzacurati et al., 2015).

## 4 Armed oHSV

A powerful means to enhance oHSV activity is to ‘arm’ it with therapeutic transgenes whose local expression in the tumor can target uninfected cancer or ‘normal’ cells. oHSV is particularly well suited for this strategy because it can incorporate large or multiple transgenes, up to ~30kb, while maintaining its life cycle. In this case, the armed oHSV also acts as a gene therapy vector. The choice of transgene is broad and includes cytokines (GM-CSF, IL-12), immunomodulatory factors (Flt3L, anti-PD1), anti-angiogenic factors (angiostatin, endostatin, vasculostatin, IL-12), TME inhibitors/degraders (chondroitinase, PTENα, MMP9, E-cadherin, ULBP3), and cytotoxic proteins (TRAIL, CYP2B1, shiCE) (Nguyen and Saha, 2021). The first and only OV approved in the US is oHSV talimogene laherparepvec (T-Vec, Imlygic™) which expresses GM-CSF (Kaufman et al., 2022). T-Vec is deleted for γ34.5 and ICP47, as in G47Δ, but there is some concern about its safety in the brain (Therapeutic Goods Administration A, 2016).

### 4.1 Cytokine transgenes

IL-12, a heterodimeric master regulator of cell-mediated immunity and an angiogenesis inhibitor, is the most potent anti-tumor cytokine expressed from oHSV (Nguyen et al., 2020). IL-12 is too toxic when delivered systemically, or in transduced tumor-infiltrating lymphocytes (TILs) (Conlon et al., 2019), thus, local expression, as with oHSV, is critical. G47Δ-mIL12 expresses murine IL-12 from the ICP6 locus of G47Δ (Cheema et al., 2013) (**Table 2**). The anti-angiogenic activity of IL-12 was examined *in vitro*, where conditioned media from G47Δ-mIL12 infected glioma cells or GSCs inhibited endothelial tube formation, and *in vivo* where it extended survival of hGSC-derived tumor-bearing mice, coincident with decreased neovasculature and VEGF expression (Zhang et al., 2013). There is a dearth of non-immunogenic mouse glioma cell lines for interrogation of immunotherapy in mouse orthotopic implant models that are representative of human GBM. Mouse 005 cells were the first murine GSC model developed (Marumoto et al., 2009). 005 mGSCs form lethal non-immunogenic orthotopic tumors that resemble human GBM in C57BL/6 mice, which present with intratumoral heterogeneity, invasiveness, neovascularity, and immunosuppressive TME (Cheema et al., 2013). This rigorous GBM model was used to evaluate the immunovirotherapeutic efficacy of G47Δ-mIL12. Intratumoral injection of G47Δ-mIL12 in 005-derived brain tumors induced a significant survival increase compared to unarmed G47Δ. This increased survival was associated with a significant reduction of tumor cells, Treg’s, and vascularity, and was dependent on T cells but not NK cells (Cheema et al., 2013).

M002 and M032 (**Figure 2**; **Table 1**) express mouse or human IL-12 respectively, from the Egr-1 promoter in a γ34.5Δ HSV (Parker et al., 2000). *In vivo*, M002 was more efficacious than its parent non-expressing R3659 in the murine syngeneic 4C8 glioma model and safe after intracerebral injection into the brains of *Aotus nancymae* primates (Markert et al., 2012). Human pediatric medulloblastoma cancer stem cells (CSCs) express high levels of nectin-1 and CD133 (Friedman et al., 2016). M002 was cytotoxic to medulloblastoma CSCs (LD50 ~ 0.5 MOI) *in vitro* and extended survival in mice with intracranial tumors to a similar extent as G207 (Friedman et al., 2016). Based on the promising M002 results, M032 was constructed for clinical translation (Roth et al., 2014). An extensive toxicology study was performed in *Aotus nancymae* primates, where it was found to be safe at 1x10^6^ pfu and 1 of 4 animals becoming moribund at the highest dose (1x10^8^ pfu) (Roth et al., 2014). Elevated white blood cells and neutrophils were observed on day 3 and then declined to baseline. Viral DNA was present in the brain, highest at the injection site, and decreased over time but still present 91 days after injection (Roth et al., 2014). Interestingly, a phase I clinical trial in dogs with glioma was performed before the human clinical trial (Omar et al., 2021). While HSV-1 is not pathogenic in dogs, canine glioma cells are similarly susceptible to killing as mouse cells. M032 treatment induced transcriptional signatures of immune modulation, both inflammatory and immunosuppressive (Chambers et al., 2021). For dogs with high-grade gliomas, the mean survival time after treatment was 108 days, and no significant adverse events were attributable to M032 (Omar et al., 2021).

R-115 expresses mouse IL-12 from the retargeted oHSV, R-LM113 (**Figure 2**; **Table 2**) (Alessandrini et al., 2019). To evaluate immune-mediated effects, a new mouse syngeneic GSC model was developed; PDGF-B transduced BALB/c neural progenitor cells were implanted orthotopically, gliomas were harvested, and isolated GSCs were transduced with human HER-2 (mHGG^pdgf^-HER2) (Reisoli et al., 2012). Infection of human and mGSCs was dependent on HER-2 expression. While R-115 did not extend the median survival of mice with mGSC-derived tumors compared to parental R-LM113, it did result in about 30% long-term survivors with none occurring in the R-LM113 group, and these were protected from rechallenge with mHGG^pdgf^ GSCs with and without HER-2 (Alessandrini et al., 2019).

OV-IL15C encodes the IL-15 complex of IL-15 and partial IL15Rα in oHSV (γ34.5Δ, ICP6^-^) (**Table 2**). IL-15 has pleiotropic roles in NK and T cell survival and activation, while the IL-15 complex with IL15Rα acts as a super-agonist. Conditioned media from infected glioma cells increases human NK or CD8+ T cell survival and killing of hGSCs (Ma et al., 2021). Treatment of orthotopic hGSC-derived tumors with hCD8+ T cells administered with OV-IL15C was significantly better than with parental OV-Q1 (Ma et al., 2021).

### 4.2 Chemokine transgene

OV-Cmab-CCL5 expresses a secreted bispecific cancer cell-targeted (anti-EGFR)-chemokine (human or mCCL5) Fc IgG1 fusion protein inserted in oHSV fHsvQuik1 (γ34.5Δ, ICP6^-^) (Tian et al., 2022) (**Table 2**). Fc IgG1 induces Fc receptor-mediated NK cytotoxicity and macrophage phagocytosis and anti-EGFR tethers CCL5-Fc to tumor cells. In a humanized mouse model with implanted EGFRvIII^+^ hGSC tumors and adoptive transfer of PBMCs and activated T cells, OV-Cmab-hCCL5 was significantly better than parental OV-Q1. In an immunocompetent GBM model, CT2A-hEGFR cells were implanted and treated with OV-Cmab-mCCL5, which induced immune cell infiltration and activation, and ‘cured’ about a third of mice when injected early after implantation. Efficacy was abolished by depleting T cells and reduced by depleting NK cells or macrophages (Tian et al., 2022). These studies illustrate how a multimodel therapy with 6 distinct activities (oHSV inflammation and cytotoxicity, chemokine activity, Fc-mediated antibody-dependent cellular cytotoxicity (ADCC) and antibody-dependent cellular phagocytosis (ADCP), and inhibition of EGFR) that target multiple cell types can ‘cure’ rigorous GBM models in mice (Sanchez Gil and Rabkin, 2022).

### 4.3 Cytotoxic transgene

oHSV-TRAIL expresses secretable TRAIL, a potent death receptor-dependent apoptosis inducer, from G47Δ (Tamura et al., 2013) (**Table 2**). GBM cells exhibit variable TRAIL resistance, whereas most cells are sensitive to oHSV, providing a rationale for oHSV-TRAIL (Tamura et al., 2013). *In vitro*, oHSV-TRAIL was more efficacious in killing temozolomide-resistant human primary and recurrent GSCs (7 of 8 lines) than oHSV (G47Δ) (Jahan et al., 2017). Intratumoral injection of oHSV-TRAIL potently inhibited the growth of human chemoresistant primary and recurrent GSC-derived intracerebral tumors through extensive induction of apoptotic cell death (Jahan et al., 2017). Thus, local expression of TRAIL from oHSV avoids systemic toxicity and overcame TMZ resistance.

### 4.4 Anti-angiogenic transgene

G47Δ-mAngio expresses murine angiostatin, which inhibited HUVEC tube formation after hGSC infection (Zhang et al., 2013) (**Table 2**). *In vivo*, G47Δ-mAngio significantly though modestly inhibited the growth of hGSC-derived brain tumors compared to G47Δ-empty, which was further improved in combination with G47Δ-mIL12, which also has anti-angiogenic activity (Zhang et al., 2013). This improved efficacy was associated with a decrease in tumor vascularity (CD31+ vessels) and VEGF expression and increased LacZ (oHSV) expression (Zhang et al., 2013). VAE is armed with an endostatin-angiostatin fusion gene inserted into a γ34.5Δ, ICP6- oHSV (Zhang et al., 2014) (**Table 2**). In an intracranial GSC-derived tumor model, VAE was significantly better than parental oHSV or recombinant endostatin at extending survival. This improvement was associated with a significant decrease in microvessel density (Zhang et al., 2014). RAMBO, a γ34.5Δ, ICP6^-^ oHSV expressing vasculostatin (extracellular fragment of BAI-1), modestly extended median survival in mice bearing intracerebral hGSC-derived tumors, which was further extended about 10% in combination with bevacizumab (anti-VEGFR) (Tomita et al., 2019) (**Table 2**).

### 4.5 Immune checkpoint transgene

Another strategy employing Fc-mediated cytotoxicity was recently reported with OV-αCD47-G1, constructed on oHSV fHsvQuik1 (γ34.5Δ, ICP6^-^) and armed with anti-CD47, an immune checkpoint that blocks phagocytosis by binding to SIRPα on myeloid cells (Xu et al., 2021) (**Table 2**). *In vitro*, conditioned media from OV-αCD47-G1 infected glioma cells significantly increased mouse myeloid cell phagocytosis, human NK cytotoxicity on co-cultured primary GBM cells from PDX tumors, and human macrophage-induced cytokine gene expression (NOS2, IL12A, IL1B) (Xu et al., 2021). Intratumoral injection of OV-αCD47-G1 in orthotopic hGSC xenografts significantly extended survival compared to parental OV-Q1, with the majority being long-term ‘cures’ (Xu et al., 2021). To test this strategy in a fully-syngeneic model, OV-A4-IgG2b was constructed with anti-mCD47 on mouse IgG2b. Intratumoral injection of OV-A4-IgG2b in CT2A tumors significantly extended survival and ‘cures’ that were dependent on macrophages (Xu et al., 2021). The results with OV-αCD47-G1 and OV-Cmab-CCL5 illustrate the power of secreted bispecific molecules interacting with Fc receptors to target both the TME and tumor cells.

### 4.6 E-cadherin transgene

oHSV efficacy can be improved by increasing virus spread in the tumor and reducing NK cell killing of infected tumor cells. OV-CDH1 was constructed on fHsvQuik1 (γ34.5Δ, ICP6^-^) and armed with human E-cadherin (CDH1) under the control of pIE4/5 promoter (Xu et al., 2018) (**Table 2**). E-cadherin binds to KLRG1 on NK cells to block NK cytolysis and cooperates with nectin-1 at cell-cell adherens junctions to facilitate oHSV infection. oHSV infection of GSCs increase their killing by NK cells, which was modestly reduced with OV-CDH1 (Xu et al., 2018). *In vivo*, OV-CDH1 injection of hGSC-derived orthotopic tumors significantly extended survival compared to parental OV-Q1, which was associated with increased virus spread and infectious virus yield, and infiltrating NK cells (Xu et al., 2018). OV-CDH1 was safe after intracerebral or intravenous injection.

## 5 Combination therapy with drugs

Successful cancer therapy is typically multimodal, requiring multiple therapeutic agents, often with different targets or activities. This is especially true for GBM due to GSCs, intratumoral heterogeneity, and immunosuppression. TMZ chemotherapy, radiation, and surgery are the standards of care for GBM patients. In addition to evaluating preclinical efficacy of oHSV combinations, it is also important to know whether combinations with standards-of-care or agents in clinical trial are detrimental. Radiation, a single low dose (5 Gy) within 24 hr of oHSV inoculation, has been combined with G207 in clinical trials for adult and pediatric gliomas, based on an increase in virus replication and postulated immune responses (Advani et al., 2011), where the combination was safe (Markert et al., 2014; Friedman et al., 2021). Studies on oHSV combination therapies targeting GSCs have mostly been limited to pharmacological agents. We have confined descriptions of oHSV combinations to those that interrogate GSCs.

### 5.1 Chemotherapy/DNA damage response

The therapeutic index for chemotherapeutic drugs is quite narrow, with severe dose-limiting toxicities, while the therapeutic index for oHSV is large with limited toxicities or resistance, and distinct cell death mechanisms. This makes the combination potentially synergistic (Kanai and Rabkin, 2013). TMZ is an oral alkylating agent that induces DNA breaks and is immune depleting. The major source of initial drug resistance in patient tumors and GSCs is the expression of MGMT, which removes the methyl guanine adducts, and is often characterized by the methylation status of the gene (Blough et al., 2010; Kanai et al., 2012a; Jahan et al., 2017). Like patients, some GSCs are resistant to TMZ (MGMT-positive) and some are sensitive. The combination of TMZ with G47Δ was synergistic in killing TMZ-sensitive hGSCs, but for TMZ-resistant GSCs was only synergistic when combined with an MGMT inhibitor O^6^-benzylguanine, while in normal human astrocytes was antagonistic (Kanai et al., 2012a). Synergy was due to oHSV-induced double-strand DNA breaks, manipulation of the DNA damage response (DDR), and sequestration of ATM (Kanai et al., 2012a). Combination treatment of TMZ-sensitive hGSC-derived orthotopic tumors was also synergistic, with about 50% long-term survivors, while a combination effect with TMZ-resistant GSCs was dependent on O^6^-benzylguanine (Kanai et al., 2012a). In contrast, when the combination of TMZ and G47Δ-mIL12 was evaluated in an immunocompetent orthotopic 005 mGSC-derived GBM model, TMZ abrogated the efficacy seen with G47Δ-mIL12 alone, even with O^6^-benzylguanine, likely due to its cytotoxic effects on tumor-infiltrating T cells and macrophages (Saha et al., 2020). This illustrates how a synergistic effect in immuno-deficient models can be reversed in an immunocompetent model and that all combinations are not beneficial.

Etoposide (VP-16), a topoisomerase II inhibitor, has been in clinical trials for GBM and pediatric brain tumors; however, high doses are very toxic. The combination of G47Δ with low doses of etoposide was moderately synergistic in killing hGSCs due to increased apoptosis (Cheema et al., 2011). In an intracerebral etoposide-resistant hGSC tumor model, the combination of low-dose etoposide with G47Δ resulted in a significant prolongation of survival compared to monotherapy that was associated with a large increase in apoptosis, as occurs *in vitro* (Cheema et al., 2011).

Poly(ADP-ribose) polymerase (PARP) plays a key role in the DDR; required for base excision repair and single-strand break repair. PARP inhibition leads to double-strand breaks (DSBs) and was found to be synthetic lethal with homologous recombination deficiency. This is the basis for the approval of PARP inhibitors (PARPis) for the treatment of BRCA1/2 mutated ovarian and breast cancer (Lord and Ashworth, 2017). Like with other therapeutics, some hGSCs are sensitive to PARPis and some are resistant, likely reflecting the tumor from which they were isolated, although all had PARP activity that was inhibited by PARPis (Ning et al., 2017). oHSVs G47Δ and MG18L synergized *in vitro* with PARPis, such as olaparib, in both sensitive and resistant hGSCs due to proteasomal degradation of Rad51 and Chk1 (Ning et al., 2017). The combination of MG18L with olaparib significantly enhanced the survival of mice bearing PARPi-resistant and -sensitive hGSC-derived tumors compared to a single agent alone, and this was associated with increased apoptosis and DSBs, and decreased Rad51 and Chk1, as *in vitro* (Ning et al., 2017).

### 5.2 Epigenetic modifiers

Epigenetic alterations regulate expression of GSC stemness and DDR (Hersh et al., 2022). oHSV infection induces innate immunity, which inhibits virus replication and spread, while histone deacetylase (HDAC) inhibitors can upregulate virus gene expression and downregulate IFN-stimulated genes (Nakashima et al., 2015b). Pretreatment of hGSCs with pan-HDAC inhibitor VPA increased rQNestin34.5 replication and cytotoxicity, and reduced associated type I IFN-responsive gene expression (Otsuki et al., 2008; Nakashima et al., 2015a). Class IIb HDAC6 deacetylates tubulin and other cytoplasmic proteins, which are involved in endocytic uptake and lysosome fusion. Virion uptake in GSCs is mostly through endocytosis. HDAC6 inhibitors tubacin and tubastatin A significantly increased virus replication in 2 of 6 hGSCs, however, there was no correlation between HDAC6 expression and GSC sensitivity (Nakashima et al., 2015a). *In vivo* analysis of rQNestin34.5 treatment of hGSC-derived orthotopic tumors found that tubastatin A significantly increased infectious virus in the tumors and extended survival, but not significantly (Nakashima et al., 2015a). Demethylating agents such as 5-azacytidine (5-Aza), also increased the replication of rQNestin34.5 in GSCs and synergized in killing GSCs, due in part to hypermethylation of the viral nestin promoter in hGSCs (Okemoto et al., 2013). The combination of rQNestin34.5 with 5-Aza or decitabine significantly extended the survival of mice with hGSC-derived tumors compared to either agent alone, including over 30% long-term survivors (Okemoto et al., 2013).

### 5.3 Molecular targeted drugs

Transforming growth factor-beta (TGFβ) plays a key role in normal development, the maintenance of GSC stemness, and reducing innate immune responses (Hersh et al., 2022). Based on the immunosuppressive properties of TGFβ, its effects on oHSV were examined. TGFβ1 treatment of NK cells rendered them less cytolytic against rQNestin34.5-infected GSCs in co-culture (Han et al., 2015). In both an hGSC-derived orthotopic xenograft and 4C8 syngeneic mouse model, pretreatment with TGFβ1 or NK cell depletion followed by rQNestin34.5 significantly extended survival, while TGFβ neutralizing antibody 1D11 abrogated oHSV efficacy (Han et al., 2015). In the immunocompetent 4C8 model, TGFβ pretreatment decreased NK and macrophage tumor infiltration and activation (Han et al., 2015). This indicated that administration of TGFβ before oHSV transiently inhibited innate immune cells, enhancing therapeutic outcomes. On the other hand, the combination of TGFβ receptor kinase inhibitor (TGFβRi) with MG18L and G47Δ synergized in killing primary and recurrent hGSCs, including TMZ-resistant (Esaki et al., 2017). Recurrent GSC TGFβR signaling, viability, and sphere formation were inhibited by TGFβRis. TGFβRi increased virus replication in recurrent hGSCs and inhibited JNK signaling, as did JNK inhibitor SP600125 (Esaki et al., 2017). Pretreatment of recurrent hGSC-derived brain tumors with TGFβR1i galunisertib followed by MG18L significantly inhibited tumor growth compared to monotherapy and resulted in about 60% long-term survivors (Esaki et al., 2017). The discordant results between the two studies could be due to the use of athymic versus SCID mice, blocking TGFβ versus TGFβR, differences between primary and recurrent hGSCs, and different oHSVs.

Integrin β1 is expressed on glioma cells, GSCs, and macrophages, and a humanized integrin β1 blocking antibody, OS2966, has shown antitumor activity. It boosted the therapeutic efficacy of rHSVQ (γ34.5Δ, ICP6^-^) through inhibition of interferon signaling and proinflammatory cytokine expression, which increased oHSV replication and cytotoxicity in hGSCs (Lee et al., 2019). In a hGSC-derived orthotopic xenograft model, OS2966 in combination with a single injection of rHSVQ significantly but modestly increased mouse survival compared to single treatments (Lee et al., 2019).

MEK kinase inhibitor trametinib is FDA-approved for BRAF-mutant melanoma. Although BRAF mutants are rare in GBM, but more frequent in pediatric gliomas, the MEK/ERK signaling pathway is often activated due to NF1 or upstream receptor kinase mutations. The combination of BRAF-mutant and MEK inhibitors has shown some clinical efficacy in BRAF-mutant gliomas (Toll et al., 2019). Trametinib treatment of macrophages co-cultured with hGSCs *in vitro* resulted in a significant reduction in tumor necrosis factor-alpha (TNF-α) secretion and an increase in rHSVQ-Luc replication in hGSCs (Yoo et al., 2019). When GSCs alone were treated, trametinib increased oHSV cytotoxicity and reduced virus replication. *In vivo*, the combination extended median survival of mice with hGSC-derived tumors modestly, in contrast curing 50% of immunodeficient mice bearing glioma cell line xenografts (Yoo et al., 2019).

Notch signaling is activated in hGSCs by oHSV infection of adjacent GSCs (inside-out signaling), which can be blocked with γ-secretase inhibitors (Otani et al., 2020). Combination treatment with oHSV 34.5ENVE of mice bearing hGSC-derived brain tumors significantly extended survival that was associated with reduced Ki67+ and increased cleaved caspase-3+ cells (Otani et al., 2020).

VEGFR tyrosine kinase inhibitor axitinib, an anti-angiogenic agent, is FDA-approved. It extended survival of immunodeficient and immunocompetent mice bearing hGSC- and mGSC-derived intracerebral tumors significantly, but modestly, which was associated with decreased vascularity (Lu et al., 2015). Mouse brain microvascular endothelial cells (MBMEC) were sensitive to axitinib, but not G47Δ-mIL12 cytotoxicity, whereas hGSCs and mGSCs (005) were sensitive to both (Saha et al., 2018). Axitinib also inhibited endothelial transdifferentiation-tube formation (Soda et al., 2011) of mGSCs but not hGSCs, while the combination with G47Δ-mIL12 reduced mGSC sphere formation, an indicator of stemness, to a greater extent than either single treatment (Saha et al., 2018). Combination treatment of recurrent hGSC xenografts with high-dose axitinib, which blocked neovascularization, and G47Δ-mIL12 significantly extended survival compared to efficacious single treatments, and this was associated with increased macrophage infiltration, tumor necrosis, and inhibition of PDGFR/ERK pathway activation (Saha et al., 2018). With the mGSC model, combination therapy was only effective in immunocompetent but not immunodeficient mice, indicating dependence on T cells, and was not enhanced with immune checkpoint inhibitors (Saha et al., 2018).

### 5.4 Immune modulatory

Immune checkpoint inhibitors (ICI) have been exceptionally effective in many solid tumors, but not GBM due to excessive immunosuppression, low tumor mutation burden (number of nonsynonymous somatic mutations (potential neoantigens) in the tumor, and ‘cold’ TME (Himes et al., 2021). Because oHSV infection induces an inflammatory TME, it is reasonable to examine whether this will overcome the resistance to ICI in GBM. In a representative orthotopic mGSC model (005), single treatments with G47Δ-mIL12, anti-PD-1, or anti-CTLA-4 had a significant but modest effect on survival, which was improved by dual combination therapy (Saha et al., 2017). Triple combination therapy acted synergistically and resulted in most mice being ‘cured’, which was associated with a decrease in CD4+ Tregs and increases in the CD8+ T/CD4+ Treg ratio, and macrophage infiltration and M1-like polarization (Saha et al., 2017). Immune cell depletion experiments revealed a complicated dependency, with CD4+ depletion completely abrogating efficacy, and CD8+ or macrophage depletion eliminating all ‘cures’ (Saha et al., 2017). Therefore, four components were necessary for curative therapy; oHSV, local IL-12 expression, and two distinct ICIs, acting with a complex interconnectedness that reflects on the challenges for GBM immunotherapy.

## 6 Conclusions and perspective

GBM is an aggressive grade 4 primary brain tumor that has a very poor prognosis, high recurrence rate, and low survival rate. The standard of care is surgical resection, radiation therapy, and TMZ chemotherapy, with a median survival of about 15 months, which hasn’t changed appreciably in decades (Nguyen et al., 2021). Some of this is due to the lack of representative models for the development of new therapies. GBM consists of a subpopulation of neoplastic cells with stem-like features including self-renewal and tumorigenicity, called GSCs (Singh et al., 2003). The identification and characterization of cancer stem cells in GBM and other cancers has revolutionized our understanding of cancer (Kreso and Dick, 2014). GSCs have been linked with tumor heterogeneity, therapy resistance, and recurrence (Prager et al., 2020); therefore, they are a critical target for therapy. They also provide important models for preclinical studies, are patient-derived, representative of the clinical condition, and embody many of the features of GBM in patients (Wakimoto et al., 2012). The mouse GSC models are an important addition to our limited armament of immunocompetent mouse models and provide more predictive *in vivo* models for new immunotherapies, including oHSV. Newer models containing GSCs are being developed; tumor organoids, 3D multicellular cultures, microfluidics, etc (Gómez-Oliva et al., 2020; Klein et al., 2020; Ruiz-Garcia et al., 2020). Coupling the models and drug efficacy with patients’ tumors and treatment outcomes will be important in understanding how predictive the models are and what features are lacking. It is clear that successfully treating GBM requires a multimodal approach targeting multiple features of the tumor.

OHSV is a novel therapeutic modality for GBM that is usually genetically-engineered for safety, selective replication, and anti-tumor activity. In contrast to other therapeutic agents, we are not aware of reports describing the development of cancer cell resistance to oHSV during therapy. OHSV is a multifaceted platform that; (i) directly kill tumor cells, amplifies itself, and spreads in the tumor, (ii) induces an inflammatory TME, anti-tumor immune responses, and behaves as an *in situ* vaccine, and (iii) can be armed with therapeutic transgenes or sequences for localized expression and activity (Jahan et al., 2021; Nguyen and Saha, 2021). With a large number of non-essential viral genes and the capacity for large sequence inserts, oHSV provides boundless opportunities for manipulation and optimization, as described in this review. Current priorities for improving oHSV efficacy and clinical outcomes include: (i) developing more potent but safe oHSV constructs; (ii) new intratumoral and systemic delivery methods; (iii) enhancing virus replication and spread in the tumor, and limiting innate antiviral responses; (iv) activating a more potent anti-tumor immune response; (v) improving TME reprogramming and remodeling; (vi) identifying synergistic interactions with other pharmacological agents; and (vii) increasing understanding of oHSV activity in patient’s tumors. Recent approval in Japan of G47Δ for the treatment of recurrent glioma has validated the use of oHSV to treat GBM (Daiichi-Sankyo, 2021) and cancer generally, and energized the field. The number of different oHSVs in clinical trials for GBM (**Table 1**) illustrates the growth and promise of oHSV immunovirotherapy.

## Author contributions

KK and JS contributed equally to this work and share first authorship. KK, JS, and SR wrote and edited the manuscript. KK and SR created the figures. All authors contributed to the article and approved the submitted version.

## Glossary

**Table:**

AC	astrocyte cell
ADCC	antibody-dependent cellular cytotoxicity
ADCP	antibody-dependent cellular phagocytosis
BBD	beclin 1 binding domain
BMP4	bone morphogenetic protein 4
CSC	cancer stem cell
DGC	differentiated glioblastoma cell
DSB	double-strand DNA break
E	early
ED50	median effective dose
EGFR	epidermal growth factor receptor
GBM	glioblastoma
GEM	genetically engineered mouse
GSC	glioblastoma stem-like cell
h	human
HGG	high-grade glioma
HDAC	histone deacetylase
m	mouse
ICI	Immune checkpoint inhibitor
IDH	isocitrate dehydrogenase
IE	immediate early
L	late
MES	mesenchymal
MGMT	O^6^-methylguanine-DNA methyltransferase
MOI	multiplicity of infection
MRI	magnetic resonance imaging
oHSV	oncolytic herpes simplex virus
OPC	oligodendrocyte progenitor cell
OV	oncolytic virus
PDX	patient-derived xenograft
PARP	poly(ADP-ribose) polymerase
pfu	plaque forming unit
RR	ribonucleotide reductase
ScGC	serum-cultured GBM cells
TGFβ	transforming growth factor-β
TGFβ receptor	TGFβ
TIL	tumor-infiltrating lymphocytes
TK	thymidine kinase
TME	tumor microenvironment
TMZ	temozolomide
Treg	T regulatory cell.

## References

[B140] Advani S. J. Markert J. M. Sood R. F. Samuel S. Gillespie G. Y. Shao M. Y.. (2011). Increased oncolytic efficacy for high-grade gliomas by optimal integration of ionizing radiation into the replicative cycle of HSV-1. Gene Ther. 18 (11), 1098–1102. doi: 10.1038/gt.2011.61 21544094 PMC8670556

[B25] Ahmed S. U. Carruthers R. Gilmour L. Yildirim S. Watts C. Chalmers A. J. (2015). Selective inhibition of parallel DNA damage response pathways optimizes radiosensitization of glioblastoma stem-like cells. Cancer Res. 75 (20), 4416–4428. doi: 10.1158/0008-5472.CAN-14-3790 26282173

[B110] Ahmed N. Salsman V. S. Kew Y. Shaffer D. Powell S. Zhang Y. J.. (2010). HER2-specific T cells target primary glioblastoma stem cells and induce regression of autologous experimental tumors. Clin. Cancer Res. 16 (2), 474–485. doi: 10.1158/1078-0432.CCR-09-1322 20068073 PMC3682507

[B87] Ahn K. Meyer T. H. Uebel S. Sempe P. Djaballah H. Yang Y.. (1996). Molecular mechanism and species specificity of TAP inhibition by herpes simplex virus ICP47. EMBO J. 15 (13), 3247–3255. doi: 10.1002/j.1460-2075.1996.tb00689.x 8670825 PMC451885

[B112] Alessandrini F. Menotti L. Avitabile E. Appolloni I. Ceresa D. Marubbi D.. (2019). Eradication of glioblastoma by immuno-virotherapy with a retargeted oncolytic HSV in a preclinical model. Oncogene 38 (23), 4467–4479. doi: 10.1038/s41388-019-0737-2 30755732

[B113] Appolloni I. Alessandrini F. Menotti L. Avitabile E. Marubbi D. Piga N.. (2021). Specificity, safety, efficacy of EGFRvIII-retargeted oncolytic HSV for xenotransplanted human glioblastoma. Viruses 13 (9), 1677. doi: 10.3390/v13091677 34578259 PMC8473268

[B24] Bao S. Wu Q. McLendon R. E. Hao Y. Shi Q. Hjelmeland A. B.. (2006). Glioma stem cells promote radioresistance by preferential activation of the DNA damage response. Nature 444 (7120), 756–760. doi: 10.1038/nature05236 17051156

[B28] Beier D. Schriefer B. Brawanski K. Hau P. Weis J. Schulz J. B.. (2012). Efficacy of clinically relevant temozolomide dosing schemes in glioblastoma cancer stem cell lines. J. Neurooncol. 109 (1), 45–52. doi: 10.1007/s11060-012-0878-4 22544650

[B32] Bhaduri A. Di Lullo E. Jung D. Müller S. Crouch E. E. Espinosa C. S.. (2020). Outer radial glia-like cancer stem cells contribute to heterogeneity of glioblastoma. Cell Stem Cell. 26 (1), 48–63.e6. doi: 10.1016/j.stem.2019.11.015 31901251 PMC7029801

[B142] Blough M. D. Westgate M. R. Beauchamp D. Kelly J. J. Stechishin O. Ramirez A. L.. (2010). Sensitivity to temozolomide in brain tumor initiating cells. Neuro Oncol. doi: 10.1093/neuonc/noq032 PMC2940656 20388697

[B62] Boehmer D. F. R. Formisano S. de Oliveira Mann C. C. Mueller S. A. Kluge M. Metzger P.. (2021). OAS1/RNase l executes RIG-I ligand-dependent tumor cell apoptosis. Sci. Immunol. 6 (61), eabe2550. doi: 10.1126/sciimmunol.abe2550 34272227

[B6] Burster T. Traut R. Yermekkyzy Z. Mayer K. Westhoff M. A. Bischof J.. (2021). Critical view of novel treatment strategies for glioblastoma: failure and success of resistance mechanisms by glioblastoma cells. Front. Cell Dev. Biol. 9, 695325. doi: 10.3389/fcell.2021.695325 34485282 PMC8415230

[B49] Calinescu A. A. Yadav V. N. Carballo E. Kadiyala P. Tran D. Zamler D. B.. (2017). Survival and proliferation of neural progenitor-derived glioblastomas under hypoxic stress is controlled by a CXCL12/CXCR4 autocrine-positive feedback mechanism. Clin. Cancer Res. 23 (5), 1250–1262. doi: 10.1158/1078-0432.CCR-15-2888 27542769 PMC5316506

[B168] Calzolari F. Appolloni I. Tutucci E. Caviglia S. Terrile M. Corte G.. (2008). Tumor progression and oncogene addiction in a PDGF-b-induced model of gliomagenesis. Neoplasia 10 (12), 1373–1382. doi: 10.1593/neo.08814 19048116 PMC2586688

[B56] Campadelli-Fiume G. Petrovic B. Leoni V. Gianni T. Avitabile E. Casiraghi C.. (2016). Retargeting strategies for oncolytic herpes simplex viruses. Viruses 8 (3), 63. doi: 10.3390/v8030063 26927159 PMC4810253

[B94] Cassady K. A. (2005). Human cytomegalovirus TRS1 and IRS1 gene products block the double-stranded-RNA-activated host protein shutoff response induced by herpes simplex virus type 1 infection. J. Virol. 79 (14), 8707–8715. doi: 10.1128/JVI.79.14.8707-8715.2005 15994764 PMC1168740

[B96] Cassady K. A. Bauer D. F. Roth J. Chambers M. R. Shoeb T. Coleman J.. (2017). Pre-clinical assessment of C134, a chimeric oncolytic herpes simplex virus, in mice and non-human primates. Mol. Ther. Oncolytics 5, 1–10. doi: 10.1016/j.omto.2017.02.001 28345027 PMC5363760

[B61] Castiello L. Sestili P. Schiavoni G. Dattilo R. Monque D. M. Ciaffoni F.. (2018). Disruption of IFN-I signaling promotes HER2/Neu tumor progression and breast cancer stem cells. Cancer Immunol. Res. 6 (6), 658–670. doi: 10.1158/2326-6066.CIR-17-0675 29622580

[B129] Chambers M. R. Foote J. B. Bentley R. T. Botta D. Crossman D. K. Della Manna D. L.. (2021). Evaluation of immunologic parameters in canine glioma patients treated with an oncolytic herpes virus. J. Transl. Genet. Genom. 5 (4), 423–442. doi: 10.20517/jtgg.2021.31 35342877 PMC8955901

[B145] Cheema T. A. Kanai R. Kim G. W. Wakimoto H. Passer B. Rabkin S. D.. (2011). Enhanced antitumor efficacy of low-dose etoposide with oncolytic herpes simplex virus in human glioblastoma stem cell xenografts. Clin. Cancer Res. 17 (23), 7383–7393. doi: 10.1158/1078-0432.CCR-11-1762 21976549 PMC3229640

[B43] Cheema T. A. Wakimoto H. Fecci P. E. Ning J. Kuroda T. Jeyaretna D. S.. (2013). Multifaceted oncolytic virus therapy for glioblastoma in an immunocompetent cancer stem cell model. Proc. Natl. Acad. Sci. U. S. A. 110 (29), 12006–12011. doi: 10.1073/pnas.1307935110 23754388 PMC3718117

[B48] Chen Z. Herting C. J. Ross J. L. Gabanic B. Puigdelloses Vallcorba M. Szulzewsky F.. (2020). Genetic driver mutations introduced in identical cell-of-origin in murine glioblastoma reveal distinct immune landscapes but similar response to checkpoint blockade. Glia 68 (10), 2148–2166. doi: 10.1002/glia.23883 32639068 PMC7512141

[B41] Cheng L. Huang Z. Zhou W. Wu Q. Donnola S. Liu J. K.. (2013). Glioblastoma stem cells generate vascular pericytes to support vessel function and tumor growth. Cell 153 (1), 139–152. doi: 10.1016/j.cell.2013.02.021 23540695 PMC3638263

[B100] Chiocca E. A. Nakashima H. Kasai K. Fernandez S. A. Oglesbee M. (2020). Preclinical toxicology of rQNestin34.5v.2: an oncolytic herpes virus with transcriptional regulation of the ICP34.5 neurovirulence gene. Mol. Ther. Methods Clin. Dev. 17, 871–893. doi: 10.1016/j.omtm.2020.03.028 32373649 PMC7195500

[B122] Conlon K. C. Miljkovic M. D. Waldmann T. A. (2019). Cytokines in the treatment of cancer. J. Interferon Cytokine Res. 39 (1), 6–21. doi: 10.1089/jir.2018.0019 29889594 PMC6350412

[B52] Costa B. Fletcher M. N. C. Boskovic P. Ivanova E. L. Eisemann T. Lohr S.. (2021). A set of cell lines derived from a genetic murine glioblastoma model recapitulates molecular and morphological characteristics of human tumors. Cancers (Basel) 13 (2), 230. doi: 10.3390/cancers13020230 33435218 PMC7827614

[B90] Daiichi-Sankyo. (2021). Daiichi sankyo launches DELYTACT® oncolytic virus G47Δ in Japan. Available at: https://www.daiichisankyo.com/files/news/pressrelease/pdf/202111/20211101_E.pdf.

[B20] Davis B. Shen Y. Poon C. C. Luchman H. A. Stechishin O. D. Pontifex C. S.. (2016). Comparative genomic and genetic analysis of glioblastoma-derived brain tumor-initiating cells and their parent tumors. Neuro Oncol. 18 (3), 350–360. doi: 10.1093/neuonc/nov143 26245525 PMC4767234

[B4] Delgado-López P. D. Corrales-García E. M. (2016). Survival in glioblastoma: a review on the impact of treatment modalities. Clin. Transl. Oncol. 18 (11), 1062–1071. doi: 10.1007/s12094-016-1497-x 26960561

[B59] Dogrammatzis C. Waisner H. Kalamvoki M. (2020). "Non-essential" proteins of HSV-1 with essential roles *In vivo*: a comprehensive review. Viruses 13 (1), 17. doi: 10.3390/v13010017 33374862 PMC7824580

[B60] Du Z. Cai C. Sims M. Boop F. A. Davidoff A. M. Pfeffer L. M. (2017). The effects of type I interferon on glioblastoma cancer stem cells. Biochem. Biophys. Res. Commun. 491 (2), 343–348. doi: 10.1016/j.bbrc.2017.07.098 28728846

[B105] Esaki S. Nigim F. Moon E. Luk S. Kiyokawa J. Curry W. Jr.. (2017). Blockade of transforming growth factor-beta signaling enhances oncolytic herpes simplex virus efficacy in patient-derived recurrent glioblastoma models. Int. J. Cancer 141 (11), 2348–2358. doi: 10.1002/ijc.30929 28801914 PMC5765440

[B26] Fouse S. D. Nakamura J. L. James C. D. Chang S. Costello J. F. (2014). Response of primary glioblastoma cells to therapy is patient specific and independent of cancer stem cell phenotype. Neuro Oncol. 16 (3), 361–371. doi: 10.1093/neuonc/not223 24311636 PMC3922516

[B84] Friedman G. K. Bernstock J. D. Chen D. Nan L. Moore B. P. Kelly V. M.. (2018). Enhanced sensitivity of patient-derived pediatric high-grade brain tumor xenografts to oncolytic HSV-1 virotherapy correlates with nectin-1 expression. Sci. Rep. 8 (1), 13930. doi: 10.1038/s41598-018-32353-x 30224769 PMC6141470

[B83] Friedman G. K. Haas M. C. Kelly V. M. Markert J. M. Gillespie G. Y. Cassady K. A. (2012). Hypoxia moderates γ(1)34.5-deleted herpes simplex virus oncolytic activity in human glioma xenoline primary cultures. Trans. Oncol. 5 (3), 200–207. doi: 10.1593/tlo.12115 PMC3384274 22741039

[B78] Friedman G. K. Johnston J. M. Bag A. K. Bernstock J. D. Li R. Aban I.. (2021). Oncolytic HSV-1 G207 immunovirotherapy for pediatric high-grade gliomas. New Engl. J. Med. 384 (17), 1613–1622. doi: 10.1056/NEJMoa2024947 33838625 PMC8284840

[B126] Friedman G. K. Moore B. P. Nan L. Kelly V. M. Etminan T. Langford C. P.. (2016). Pediatric medulloblastoma xenografts including molecular subgroup 3 and CD133+ and CD15+ cells are sensitive to killing by oncolytic herpes simplex viruses. Neuro Oncol. 18 (2), 227–235. doi: 10.1093/neuonc/nov123 26188016 PMC4724175

[B95] Friedman G. K. Nan L. Haas M. C. Kelly V. M. Moore B. P. Langford C. P.. (2015). γ_1_34.5-deleted HSV-1-expressing human cytomegalovirus IRS1 gene kills human glioblastoma cells as efficiently as wild-type HSV-1 in normoxia or hypoxia. Gene Ther. 22 (4), 348–355. doi: 10.1038/gt.2014.107 25427614 PMC4383690

[B33] Galdieri L. Jash A. Malkova O. Mao D. D. DeSouza P. Chu Y. E.. (2021). Defining phenotypic and functional heterogeneity of glioblastoma stem cells by mass cytometry. JCI Insight 6 (4), e128456. doi: 10.1172/jci.insight.128456 33400685 PMC7934942

[B111] Gambini E. Reisoli E. Appolloni I. Gatta V. Campadelli-Fiume G. Menotti L.. (2012). Replication-competent herpes simplex virus retargeted to HER2 as therapy for high-grade glioma. Mol. Ther. 20 (5), 994–1001. doi: 10.1038/mt.2012.22 22354378 PMC3345974

[B115] Gaur A. Jewell D. A. Liang Y. Ridzon D. Moore J. H. Chen C.. (2007). Characterization of microRNA expression levels and their biological correlates in human cancer cell lines. Cancer Res. 67 (6), 2456–2468. doi: 10.1158/0008-5472.CAN-06-2698 17363563

[B55] Goins W. F. Hall B. Cohen J. B. Glorioso J. C. (2016). Retargeting of herpes simplex virus (HSV) vectors. Curr. Opin. Virol. 21, 93–101. doi: 10.1016/j.coviro.2016.08.007 27614209 PMC5138136

[B161] Gómez-Oliva R. Domínguez-García S. Carrascal L. Abalos-Martínez J. Pardillo-Díaz R. Verástegui C.. (2020). Evolution of experimental models in the study of glioblastoma: toward finding efficient treatments. Front. Oncol. 10, 614295. doi: 10.3389/fonc.2020.614295 33585240 PMC7878535

[B151] Han J. Chen X. Chu J. Xu B. Meisen W. H. Chen L.. (2015). TGFβ treatment enhances glioblastoma virotherapy by inhibiting the innate immune response. Cancer Res. 75 (24), 5273–5282. doi: 10.1158/0008-5472.CAN-15-0894 26631269 PMC4681611

[B68] Harrow S. Papanastassiou V. Harland J. Mabbs R. Petty R. Fraser M.. (2004). HSV1716 injection into the brain adjacent to tumour following surgical resection of high-grade glioma: safety data and long-term survival. Gene Ther. 11 (22), 1648–1658. doi: 10.1038/sj.gt.3302289 15334111

[B107] Hersh A. M. Gaitsch H. Alomari S. Lubelski D. Tyler B. M. (2022). Molecular pathways and genomic landscape of glioblastoma stem cells: opportunities for targeted therapy. Cancers (Basel) 14 (15), 3743. doi: 10.3390/cancers14153743 35954407 PMC9367289

[B159] Himes B. T. Geiger P. A. Ayasoufi K. Bhargav A. G. Brown D. A. Parney I. F. (2021). Immunosuppression in glioblastoma: current understanding and therapeutic implications. Front. Oncol. 11, 770561. doi: 10.3389/fonc.2021.770561 34778089 PMC8581618

[B65] Jahan N. Ghouse S. M. Martuza R. L. Rabkin S. D. (2021). *In situ* cancer vaccination and immunovirotherapy using oncolytic HSV. Viruses 13 (9), 1740. doi: 10.3390/v13091740 34578321 PMC8473045

[B135] Jahan N. Lee J. M. Shah K. Wakimoto H. (2017). Therapeutic targeting of chemoresistant and recurrent glioblastoma stem cells with a proapoptotic variant of oncolytic herpes simplex virus. Int. J. Cancer 141 (8), 1671–1681. doi: 10.1002/ijc.30811 28567859 PMC5796532

[B51] Jin F. Jin-Lee H. J. Johnson A. J. (2021). “Mouse models of experimental glioblastoma,” in Gliomas. Ed. Debinski W. (Brisbane (AU: Exon Publications Copyright). 34038046

[B97] Kambara H. Okano H. Chiocca E. A. Saeki Y. (2005). An oncolytic HSV-1 mutant expressing ICP34.5 under control of a nestin promoter increases survival of animals even when symptomatic from a brain tumor. Cancer Res. 65 (7), 2832–2839. doi: 10.1158/0008-5472.CAN-04-3227 15805284

[B141] Kanai R. Rabkin S. D. (2013). Combinatorial strategies for oncolytic herpes simplex virus therapy of brain tumors. CNS Oncol. 2 (2), 129–142. doi: 10.2217/cns.12.42 23687568 PMC3655794

[B143] Kanai R. Rabkin S. D. Yip S. Sgubin D. Zaupa C. M. Hirose Y.. (2012a). Oncolytic virus-mediated manipulation of DNA damage responses: synergy with chemotherapy in killing glioblastoma stem cells. J. Natl. Cancer Inst. 104 (1), 42–55. doi: 10.1093/jnci/djr509 22173583 PMC3250384

[B102] Kanai R. Wakimoto H. Martuza R. L. Rabkin S. D. (2011). A novel oncolytic herpes simplex virus that synergizes with phosphoinositide 3-kinase/Akt pathway inhibitors to target glioblastoma stem cells. Clin. Cancer Res. 17 (11), 3686–3696. doi: 10.1158/1078-0432.CCR-10-3142 21505062 PMC3107877

[B80] Kanai R. Zaupa C. Sgubin D. Antoszczyk S. J. Martuza R. L. Wakimoto H.. (2012b). Effect of gamma34.5 deletions on oncolytic herpes simplex virus activity in brain tumors. J. Virol. 86 (8), 4420–4431. doi: 10.1128/JVI.00017-12 22345479 PMC3318611

[B58] Kangas C. Krawczyk E. He B. (2021). Oncolytic HSV: underpinnings of tumor susceptibility. Viruses 13 (7), 1408. doi: 10.3390/v13071408 34372614 PMC8310378

[B119] Kaufman H. L. Shalhout S. Z. Iodice G. (2022). Talimogene laherparepvec: moving from first-In-Class to best-In-Class. Front. Mol. Biosci. 9, 834841. doi: 10.3389/fmolb.2022.834841 35274007 PMC8901478

[B117] Kennedy E. M. Farkaly T. Grzesik P. Lee J. Denslow A. Hewett J.. (2020). Design of an interferon-resistant oncolytic HSV-1 incorporating redundant safety modalities for improved tolerability. Mol. Ther. Oncolytics 18, 476–490. doi: 10.1016/j.omto.2020.08.004 32953982 PMC7479328

[B47] Khalsa J. K. Cheng N. Keegan J. Chaudry A. Driver J. Bi W. L.. (2020). Immune phenotyping of diverse syngeneic murine brain tumors identifies immunologically distinct types. Nat. Commun. 11 (1), 3912. doi: 10.1038/s41467-020-17704-5 32764562 PMC7411074

[B162] Klein E. Hau A. C. Oudin A. Golebiewska A. Niclou S. P. (2020). Glioblastoma organoids: pre-clinical applications and challenges in the context of immunotherapy. Front. Oncol. 10, 604121. doi: 10.3389/fonc.2020.604121 33364198 PMC7753120

[B14] Kreso A. Dick J. E. (2014). Evolution of the cancer stem cell model. Cell Stem Cell. 14 (3), 275–291. doi: 10.1016/j.stem.2014.02.006 24607403

[B13] Lapidot T. Sirard C. Vormoor J. Murdoch B. Hoang T. Caceres-Cortes J.. (1994). A cell initiating human acute myeloid leukaemia after transplantation into SCID mice. Nature 367 (6464), 645–648. doi: 10.1038/367645a0 7509044

[B17] Lee J. Kotliarova S. Kotliarov Y. Li A. Su Q. Donin N. M.. (2006). Tumor stem cells derived from glioblastomas cultured in bFGF and EGF more closely mirror the phenotype and genotype of primary tumors than do serum-cultured cell lines. Cancer Cell. 9 (5), 391–403. doi: 10.1016/j.ccr.2006.03.030 16697959

[B152] Lee T. J. Nair M. Banasavadi-Siddegowda Y. Liu J. Nallanagulagari T. Jaime-Ramirez A. C.. (2019). Enhancing therapeutic efficacy of oncolytic herpes simplex virus-1 with integrin β1 blocking antibody OS2966. Mol. Cancer Ther. 18 (6), 1127–1136. doi: 10.1158/1535-7163.MCT-18-0953 30926634 PMC6548661

[B103] Leopardi R. Van Sant C. Roizman B. (1997). The herpes simplex virus 1 protein kinase US3 is required for protection from apoptosis induced by the virus. Proc. Natl. Acad. Sci. U.S.A. 94 (15), 7891–7896. doi: 10.1073/pnas.94.15.7891 9223283 PMC21525

[B146] Lord C. J. Ashworth A. (2017). PARP inhibitors: synthetic lethality in the clinic. Science 355 (6330), 1152–1158. doi: 10.1126/science.aam7344 28302823 PMC6175050

[B2] Louis D. N. Perry A. Wesseling P. Brat D. J. Cree I. A. Figarella-Branger D.. (2021). The 2021 WHO classification of tumors of the central nervous system: a summary. Neuro Oncol. 23 (8), 1231–1251. doi: 10.1093/neuonc/noab106 34185076 PMC8328013

[B156] Lu L. Saha D. Martuza R. L. Rabkin S. D. Wakimoto H. (2015). Single agent efficacy of the VEGFR kinase inhibitor axitinib in preclinical models of glioblastoma. J. Neurooncol. 121 (1), 91–100. doi: 10.1007/s11060-014-1612-1 25213669 PMC4751887

[B98] Ludwig K. Kornblum H. I. (2017). Molecular markers in glioma. J. Neurooncol. 134 (3), 505–512. doi: 10.1007/s11060-017-2379-y 28233083 PMC5568999

[B131] Ma R. Lu T. Li Z. Teng K. Y. Mansour A. G. Yu M.. (2021). An oncolytic virus expressing IL15/IL15Rα combined with off-the-Shelf EGFR-CAR NK cells targets glioblastoma. Cancer Res. 81 (13), 3635–3648. doi: 10.1158/0008-5472.CAN-21-0035 34006525 PMC8562586

[B164] MacLean A. R. Ul-Fareed M. Robertson L. Harland J. Brown S. M. (1991). Herpes simplex virus type 1 deletion variants 1714 and 1716 pinpoint neurovirulence-related sequences in Glasgow strain 17+ between immediate early gene 1 and the 'a' sequence. J. Gen. Virol. 72 (Pt 3), 631–639. doi: 10.1099/0022-1317-72-3-631 1848598

[B125] Markert J. M. Cody J. J. Parker J. N. Coleman J. M. Price K. H. Kern E. R.. (2012). Preclinical evaluation of a genetically engineered herpes simplex virus expressing interleukin-12. J. Virol. 86 (9), 5304–5313. doi: 10.1128/JVI.06998-11 22379082 PMC3347348

[B76] Markert J. M. Liechty P. G. Wang W. Gaston S. Braz E. Karrasch M.. (2009). Phase ib trial of mutant herpes simplex virus G207 inoculated pre-and post-tumor resection for recurrent GBM. Mol. Ther. 17 (1), 199–207. doi: 10.1038/mt.2008.228 18957964 PMC2834981

[B75] Markert J. M. Medlock M. D. Rabkin S. D. Gillespie G. Y. Todo T. Hunter W. D.. (2000). Conditionally replicating herpes simplex virus mutant, G207 for the treatment of malignant glioma: results of a phase I trial. Gene Ther. 7 (10), 867–874. doi: 10.1038/sj.gt.3301205 10845725

[B77] Markert J. M. Razdan S. N. Kuo H. C. Cantor A. Knoll A. Karrasch M.. (2014). A phase 1 trial of oncolytic HSV-1, G207, given in combination with radiation for recurrent GBM demonstrates safety and radiographic responses. Mol. Ther. 22 (5), 1048–1055. doi: 10.1038/mt.2014.22 24572293 PMC4015243

[B57] Martuza R. L. Malick A. Markert J. M. Ruffner K. L. Coen D. M. (1991). Experimental therapy of human glioma by means of a genetically engineered virus mutant. Science 252 (5007), 854–856. doi: 10.1126/science.1851332 1851332

[B46] Marumoto T. Tashiro A. Friedmann-Morvinski D. Scadeng M. Soda Y. Gage F. H.. (2009). Development of a novel mouse glioma model using lentiviral vectors. Nat. Med. 15 (1), 110–116. doi: 10.1038/nm.1863 19122659 PMC2671237

[B116] Mazzacurati L. Marzulli M. Reinhart B. Miyagawa Y. Uchida H. Goins W. F.. (2015). Use of miRNA response sequences to block off-target replication and increase the safety of an unattenuated, glioblastoma-targeted oncolytic HSV. Mol. Ther. 23 (1), 99–107. doi: 10.1038/mt.2014.177 25200130 PMC4426800

[B169] Mazzoleni S. Politi L. S. Pala M. Cominelli M. Franzin A. Sergi Sergi L.. (2010). Epidermal growth factor receptor expression identifies functionally and molecularly distinct tumor-initiating cells in human glioblastoma multiforme and is required for gliomagenesis. Cancer Res. 70 (19), 7500–7513. doi: 10.1158/0008-5472.CAN-10-2353 20858720

[B109] Menotti L. Cerretani A. Hengel H. Campadelli-Fiume G. (2008). Construction of a fully retargeted herpes simplex virus 1 recombinant capable of entering cells solely *via* human epidermal growth factor receptor 2. J. Virol. 82 (20), 10153–10161. doi: 10.1128/JVI.01133-08 18684832 PMC2566291

[B166] Miller K. E. Cassady K. A. Roth J. C. Clements J. Schieffer K. M. Leraas K.. (2022). Immune activity and response differences of oncolytic viral therapy in recurrent glioblastoma: gene expression analyses of a phase IB study. Clin. Cancer Res. 28 (3), 498–506. doi: 10.1158/1078-0432.CCR-21-2636 35105718 PMC8846434

[B71] Mineta T. Rabkin S. D. Martuza R. L. (1994). Treatment of malignant gliomas using ganciclovir-hypersensitive, ribonucleotide reductase-deficient herpes simplex viral mutant. Cancer Res. 54 (15), 3963–3966. 8033122

[B72] Mineta T. Rabkin S. D. Yazaki T. Hunter W. D. Martuza R. L. (1995). Attenuated multi-mutated herpes simplex virus-1 for the treatment of malignant gliomas. Nat. Med. 1 (9), 938–943. doi: 10.1038/nm0995-938 7585221

[B85] Mohr I. Gluzman Y. (1996). A herpesvirus genetic element which affects translation in the absence of the viral GADD34 function. EMBO J. 15 (17), 4759–4766. doi: 10.1002/j.1460-2075.1996.tb00853.x 8887567 PMC452208

[B86] Mulvey M. Poppers J. Ladd A. Mohr I. (1999). A herpesvirus ribosome-associated, RNA-binding protein confers a growth advantage upon mutants deficient in a GADD34-related function. J. Virol. 73 (4), 3375–3385. doi: 10.1128/JVI.73.4.3375-3385.1999 10074192 PMC104102

[B63] Musella M. Galassi C. Manduca N. Sistigu A. (2021). The yin and yang of type I IFNs in cancer promotion and immune activation. Biol. (Basel) 10 (9), 856. doi: 10.3390/biology10090856 PMC8467547 34571733

[B149] Nakashima H. Kaufmann J. K. Wang P. Y. Nguyen T. Speranza M. C. Kasai K.. (2015a). Histone deacetylase 6 inhibition enhances oncolytic viral replication in glioma. J. Clin. Invest. 125 (11), 4269–4280. doi: 10.1172/JCI80713 26524593 PMC4639993

[B147] Nakashima H. Nguyen T. Chiocca E. A. (2015b). Combining HDAC inhibitors with oncolytic virotherapy for cancer therapy. Oncolytic Virother. 4, 183–191. doi: 10.2147/OV.S66081 27512681 PMC4918398

[B101] Nakashima H. Nguyen T. Kasai K. Passaro C. Ito H. Goins W. F.. (2018). Toxicity and efficacy of a novel GADD34-expressing oncolytic HSV-1 for the treatment of experimental glioblastoma. Clin. Cancer Res. 24 (11), 2574–2584. doi: 10.1158/1078-0432.CCR-17-2954 29511029 PMC6800093

[B42] Neftel C. Laffy J. Filbin M. G. Hara T. Shore M. E. Rahme G. J.. (2019). An integrative model of cellular states, plasticity, and genetics for glioblastoma. Cell 178 (4), 835–49.e21. doi: 10.1016/j.cell.2019.06.024 31327527 PMC6703186

[B5] Nguyen H. M. Guz-Montgomery K. Lowe D. B. Saha D. (2021). Pathogenetic features and current management of glioblastoma. Cancers (Basel) 13 (4). doi: 10.3390/cancers13040856 PMC7922739 33670551

[B121] Nguyen H. M. Guz-Montgomery K. Saha D. (2020). Oncolytic virus encoding a master pro-inflammatory cytokine interleukin 12 in cancer immunotherapy. Cells 9 (2), 400. doi: 10.3390/cells9020400 32050597 PMC7072539

[B118] Nguyen H. M. Saha D. (2021). The current state of oncolytic herpes simplex virus for glioblastoma treatment. Oncolytic Virother. 10, 1–27. doi: 10.2147/OV.S268426 33659221 PMC7917312

[B19] Nigim F. Cavanaugh J. Patel A. P. Curry W. T. Jr. Esaki S. Kasper E. M.. (2015). Targeting hypoxia-inducible factor 1alpha in a new orthotopic model of glioblastoma recapitulating the hypoxic tumor microenvironment. J. Neuropathol. Exp. Neurol. 74 (7), 710–722. doi: 10.1097/NEN.0000000000000210 26083570 PMC4473779

[B29] Ning J. F. Stanciu M. Humphrey M. R. Gorham J. Wakimoto H. Nishihara R.. (2019). Myc targeted CDK18 promotes ATR and homologous recombination to mediate PARP inhibitor resistance in glioblastoma. Nat. Commun. 10 (1), 2910. doi: 10.1038/s41467-019-10993-5 31266951 PMC6606647

[B106] Ning J. Wakimoto H. Peters C. Martuza R. L. Rabkin S. D. (2017). Rad51 degradation: role in oncolytic virus-Poly(ADP-Ribose) polymerase inhibitor combination therapy in glioblastoma. J. Natl. Cancer Inst. 109 (3), 1–13. doi: 10.1093/jnci/djw229 PMC6059185 28376211

[B150] Okemoto K. Kasai K. Wagner B. Haseley A. Meisen H. Bolyard C.. (2013). DNA Demethylating agents synergize with oncolytic HSV1 against malignant gliomas. Clin. Cancer Res. 19 (21), 5952–5959. doi: 10.1158/1078-0432.CCR-12-3588 24056786 PMC3860592

[B128] Omar N. B. Bentley R. T. Crossman D. K. Foote J. B. Koehler J. W. Markert J. M.. (2021). Safety and interim survival data after intracranial administration of M032, a genetically engineered oncolytic HSV-1 expressing IL-12, in pet dogs with sporadic gliomas. Neurosurg. Focus. 50 (2), E5. doi: 10.3171/2020.11.FOCUS20844 PMC8383155 33524948

[B93] Orvedahl A. Alexander D. Talloczy Z. Sun Q. Wei Y. Zhang W.. (2007). HSV-1 ICP34.5 confers neurovirulence by targeting the beclin 1 autophagy protein. Cell Host Microbe 1 (1), 23–35. doi: 10.1016/j.chom.2006.12.001 18005679

[B1] Ostrom Q. T. Patil N. Cioffi G. Waite K. Kruchko C. Barnholtz-Sloan J. S. (2020). CBTRUS statistical report: primary brain and other central nervous system tumors diagnosed in the united states in 2013-2017. Neuro Oncol. 22 (12 Suppl 2), iv1–iv96. doi: 10.1093/neuonc/noaa200 33123732 PMC7596247

[B155] Otani Y. Yoo J. Y. Chao S. Liu J. Jaime-Ramirez A. C. Lee T. J.. (2020). Oncolytic HSV-infected glioma cells activate NOTCH in adjacent tumor cells sensitizing tumors to gamma secretase inhibition. Clin. Cancer Res. 26 (10), 2381–2392. doi: 10.1158/1078-0432.CCR-19-3420 32139403 PMC7325527

[B148] Otsuki A. Patel A. Kasai K. Suzuki M. Kurozumi K. Chiocca E. A.. (2008). Histone deacetylase inhibitors augment antitumor efficacy of herpes-based oncolytic viruses. Mol. Ther. 16 (9), 1546–1555. doi: 10.1038/mt.2008.155 18648350

[B67] Papanastassiou V. Rampling R. Fraser M. Petty R. Hadley D. Nicoll J.. (2002). The potential for efficacy of the modified (ICP 34.5(-)) herpes simplex virus HSV1716 following intratumoural injection into human malignant glioma: a proof of principle study. Gene Ther. 9 (6), 398–406. doi: 10.1038/sj.gt.3301664 11960316

[B124] Parker J. N. Gillespie G. Y. Love C. E. Randall S. Whitley R. J. Markert J. M. (2000). Engineered herpes simplex virus expressing IL-12 in the treatment of experimental murine brain tumors. Proc. Natl. Acad. Sci. U.S.A. 97 (5), 2208–2213. doi: 10.1073/pnas.040557897 10681459 PMC15779

[B104] Parsons D. W. Jones S. Zhang X. Lin J. C. Leary R. J. Angenendt P.. (2008). An integrated genomic analysis of human glioblastoma multiforme. Science 321 (5897), 1807–1812. doi: 10.1126/science.1164382 18772396 PMC2820389

[B167] Patel D. M. Foreman P. M. Nabors L. B. Riley K. O. Gillespie G. Y. Markert J. M. (2016). Design of a phase I clinical trial to evaluate M032, a genetically engineered HSV-1 expressing IL-12, in patients with Recurrent/Progressive glioblastoma multiforme, anaplastic astrocytoma, or gliosarcoma. Hum. Gene Ther. Clin. Dev. 27 (2), 69–78. doi: 10.1089/humc.2016.031 27314913 PMC4932657

[B8] Persico P. Lorenzi E. Dipasquale A. Pessina F. Navarria P. Politi L. S.. (2021). Checkpoint inhibitors as high-grade gliomas treatment: state of the art and future perspectives. J. Clin. Med. 10 (7). doi: 10.3390/jcm10071367 PMC8036455 33810532

[B21] Pesenti C. Navone S. E. Guarnaccia L. Terrasi A. Costanza J. Silipigni R.. (2019). The genetic landscape of human glioblastoma and matched primary cancer stem cells reveals intratumour similarity and intertumour heterogeneity. Stem Cells Int. 2019, 2617030. doi: 10.1155/2019/2617030 30984267 PMC6431486

[B82] Peters C. Paget M. Tshilenge K. T. Saha D. Antoszczyk S. Baars A.. (2018). Restriction of replication of oncolytic herpes simplex virus with a deletion of gamma34.5 in glioblastoma stem-like cells. J. Virol. 92 (15), e00246–e00218. doi: 10.1128/JVI.00246-18 29793956 PMC6052301

[B53] Peters C. Rabkin S. D. (2015). Designing herpes viruses as oncolytics. Mol. Ther. Oncolytics 2, 15010. doi: 10.1038/mto.2015.10 26462293 PMC4599707

[B34] Piccirillo S. G. Reynolds B. A. Zanetti N. Lamorte G. Binda E. Broggi G.. (2006). Bone morphogenetic proteins inhibit the tumorigenic potential of human brain tumour-initiating cells. Nature 444 (7120), 761–765. doi: 10.1038/nature05349 17151667

[B11] Prager B. C. Bhargava S. Mahadev V. Hubert C. G. Rich J. N. (2020). Glioblastoma stem cells: driving resilience through chaos. Trends Cancer 6 (3), 223–235. doi: 10.1016/j.trecan.2020.01.009 32101725 PMC8779821

[B69] Pyles R. B. Warnick R. E. Chalk C. L. Szanti B. E. Parysek L. M. (1997). A novel multiply-mutated HSV-1 strain for the treatment of human brain tumors. Hum. Gene Ther. 8 (5), 533–544. doi: 10.1089/hum.1997.8.5-533 9095405

[B165] Rampling R. Cruickshank G. Papanastassiou V. Nicoll J. Hadley D. Brennan D.. (2000). Toxicity evaluation of replication-competent herpes simplex virus (ICP 34.5 null mutant 1716) in patients with recurrent malignant glioma. Gene Ther. 7 (10), 859–866. doi: 10.1038/sj.gt.3301184 10845724

[B130] Reisoli E. Gambini E. Appolloni I. Gatta V. Barilari M. Menotti L.. (2012). Efficacy of HER2 retargeted herpes simplex virus as therapy for high-grade glioma in immunocompetent mice. Cancer Gene Ther. 19 (11), 788–795. doi: 10.1038/cgt.2012.62 22996742

[B39] Ricci-Vitiani L. Pallini R. Biffoni M. Todaro M. Invernici G. Cenci T.. (2010). Tumour vascularization *via* endothelial differentiation of glioblastoma stem-like cells. Nature 468 (7325), 824–828. doi: 10.1038/nature09557 21102434

[B54] Roizman B. Zhou G. (2015). The 3 facets of regulation of herpes simplex virus gene expression: a critical inquiry. Virology 479-480, 562–567. doi: 10.1016/j.virol.2015.02.036 25771487 PMC4424108

[B23] Rosenberg S. Verreault M. Schmitt C. Guegan J. Guehennec J. Levasseur C.. (2017). Multi-omics analysis of primary glioblastoma cell lines shows recapitulation of pivotal molecular features of parental tumors. Neuro Oncol. 19 (2), 219–228. doi: 10.1093/neuonc/now160 27571888 PMC5463853

[B127] Roth J. C. Cassady K. A. Cody J. J. Parker J. N. Price K. H. Coleman J. M.. (2014). Evaluation of the safety and biodistribution of M032, an attenuated herpes simplex virus type 1 expressing hIL-12, after intracerebral administration to aotus nonhuman primates. Hum. Gene Ther. Clin. Dev. 25 (1), 16–27. doi: 10.1089/humc.2013.201 24649838 PMC4047998

[B45] Rousso-Noori L. Mastandrea I. Talmor S. Waks T. Globerson Levin A. Haugas M.. (2021). P32-specific CAR T cells with dual antitumor and antiangiogenic therapeutic potential in gliomas. Nat. Commun. 12 (1), 3615. doi: 10.1038/s41467-021-23817-2 34127674 PMC8203650

[B163] Ruiz-Garcia H. Alvarado-Estrada K. Schiapparelli P. Quinones-Hinojosa A. Trifiletti D. M. (2020). Engineering three-dimensional tumor models to study glioma cancer stem cells and tumor microenvironment. Front. Cell Neurosci. 14, 558381. doi: 10.3389/fncel.2020.558381 33177991 PMC7596188

[B9] Saha D. Ahmed S. S. Rabkin S. D. (2015). Exploring the antitumor effect of virus in malignant glioma. Drugs Future 40 (11), 739–749. doi: 10.1358/dof.2015.040.11.2383070 26855472 PMC4743035

[B160] Saha D. Martuza R. L. Rabkin S. D. (2017). Macrophage polarization contributes to glioblastoma eradication by combination immunovirotherapy and immune checkpoint blockade. Cancer Cell. 32 (2), 253–267. doi: 10.1016/j.ccell.2017.07.006 28810147 PMC5568814

[B144] Saha D. Rabkin S. D. Martuza R. L. (2020). Temozolomide antagonizes oncolytic immunovirotherapy in glioblastoma. J. Immunother. Cancer 8 (1), e000345. doi: 10.1136/jitc-2019-000345 32457126 PMC7252967

[B157] Saha D. Wakimoto H. Peters C. W. Antoszczyk S. J. Rabkin S. D. Martuza R. L. (2018). Combinatorial effects of VEGFR kinase inhibitor axitinib and oncolytic virotherapy in mouse and human glioblastoma stem-like cell models. Clin. Cancer Res. 24 (14), 3409–3422. doi: 10.1158/1078-0432.CCR-17-1717 29599413 PMC6050085

[B114] Sanchez Gil J. Dubois M. Neirinckx V. Lombard A. Coppieters N. D'Arrigo P.. (2022). Nanobody-based retargeting of an oncolytic herpesvirus for eliminating CXCR4(+) GBM cells: a proof of principle. Mol. Ther. Oncolytics 26, 35–48. doi: 10.1016/j.omto.2022.06.002 35784400 PMC9217993

[B133] Sanchez Gil J. Rabkin S. D. (2022). An armed oncolytic virus for GBM destruction. Nat. Cancer 3 (11), 1274–1276. doi: 10.1038/s43018-022-00457-z 36396753

[B27] Segerman A. Niklasson M. Haglund C. Bergström T. Jarvius M. Xie Y.. (2016). Clonal variation in drug and radiation response among glioma-initiating cells is linked to proneural-mesenchymal transition. Cell Rep. 17 (11), 2994–3009. doi: 10.1016/j.celrep.2016.11.056 27974212

[B81] Shah A. C. Parker J. N. Gillespie G. Y. Lakeman F. D. Meleth S. Markert J. M.. (2007). Enhanced antiglioma activity of chimeric HCMV/HSV-1 oncolytic viruses. Gene Ther. 14 (13), 1045–1054. doi: 10.1038/sj.gt.3302942 17429445

[B22] Shen Y. Grisdale C. J. Islam S. A. Bose P. Lever J. Zhao E. Y.. (2019). Comprehensive genomic profiling of glioblastoma tumors, BTICs, and xenografts reveals stability and adaptation to growth environments. Proc. Natl. Acad. Sci. U.S.A. 116 (38), 19098–19108. doi: 10.1073/pnas.1813495116 31471491 PMC6754609

[B15] Singh S. K. Clarke I. D. Terasaki M. Bonn V. E. Hawkins C. Squire J.. (2003). Identification of a cancer stem cell in human brain tumors. Cancer Res. 63 (18), 5821–5828. 14522905

[B16] Singh S. K. Hawkins C. Clarke I. D. Squire J. A. Bayani J. Hide T.. (2004). Identification of human brain tumour initiating cells. Nature 432 (7015), 396–401. doi: 10.1038/nature03128 15549107

[B158] Soda Y. Marumoto T. Friedmann-Morvinski D. Soda M. Liu F. Michiue H.. (2011). Transdifferentiation of glioblastoma cells into vascular endothelial cells. Proc. Natl. Acad. Sci. U.S.A. 108 (11), 4274–4280. doi: 10.1073/pnas.1016030108 21262804 PMC3060261

[B64] Solomon P. E. Kirkemo L. L. Wilson G. M. Leung K. K. Almond M. H. Sayles L. C.. (2022). Discovery proteomics analysis determines that driver oncogenes suppress antiviral defense pathways through reduction in interferon-β autocrine stimulation. Mol. Cell. Proteomics 21 (7), 100247. doi: 10.1016/j.mcpro.2022.100247 35594991 PMC9212846

[B7] Sottoriva A. Spiteri I. Piccirillo S. G. Touloumis A. Collins V. P. Marioni J. C.. (2013). Intratumor heterogeneity in human glioblastoma reflects cancer evolutionary dynamics. Proc. Natl. Acad. Sci. U.S.A. 110 (10), 4009–4014. doi: 10.1073/pnas.1219747110 23412337 PMC3593922

[B3] Stupp R. Hegi M. E. Mason W. P. van den Bent M. J. Taphoorn M. J. Janzer R. C.. (2009). Effects of radiotherapy with concomitant and adjuvant temozolomide versus radiotherapy alone on survival in glioblastoma in a randomised phase III study: 5-year analysis of the EORTC-NCIC trial. Lancet Oncol. 10 (5), 459–466. doi: 10.1016/S1470-2045(09)70025-7 19269895

[B91] Stupp R. Taillibert S. Kanner A. Read W. Steinberg D. Lhermitte B.. (2017). Effect of tumor-treating fields plus maintenance temozolomide vs maintenance temozolomide alone on survival in patients with glioblastoma: a randomized clinical trial. Jama 318 (23), 2306–2316. doi: 10.1001/jama.2017.18718 29260225 PMC5820703

[B36] Suva M. L. Rheinbay E. Gillespie S. M. Patel A. P. Wakimoto H. Rabkin S. D.. (2014). Reconstructing and reprogramming the tumor-propagating potential of glioblastoma stem-like cells. Cell 157 (3), 580–594. doi: 10.1016/j.cell.2014.02.030 24726434 PMC4004670

[B31] Suvà M. L. Tirosh I. (2020). The glioma stem cell model in the era of single-cell genomics. Cancer Cell. 37 (5), 630–636. doi: 10.1016/j.ccell.2020.04.001 32396858

[B92] Talloczy Z. HWt V. Levine B. (2006). PKR-dependent autophagic degradation of herpes simplex virus type 1. Autophagy 2 (1), 24–29. doi: 10.4161/auto.2176 16874088

[B134] Tamura K. Wakimoto H. Agarwal A. S. Rabkin S. D. Bhere D. Martuza R. L.. (2013). Multimechanistic tumor targeted oncolytic virus overcomes resistance in brain tumors. Mol. Ther. 21 (1), 68–77. doi: 10.1038/mt.2012.175 22929661 PMC3538303

[B30] Tanaka S. Luk S. Kiyokawa J. Onozato M. L. Iafrate A. J. Shah K.. (2019). Genetically distinct glioma stem-like cell xenografts established from paired glioblastoma samples harvested before and after molecularly targeted therapy. Sci. Rep. 9 (1), 139. doi: 10.1038/s41598-018-37437-2 30644426 PMC6333836

[B120] Therapeutic Goods Administration A. (2016). Australian Public assessment report for talimogene laherparepvec 2016. Available at: https://www.tga.gov.au/sites/default/files/auspar-talimogene-laherparepvec-160531.pdf.

[B132] Tian L. Xu B. Chen Y. Li Z. Wang J. Zhang J.. (2022). Specific targeting of glioblastoma with an oncolytic virus expressing a cetuximab-CCL5 fusion protein via innate and adaptive immunity. Nat. Cancer 3, 1318–1335. doi: 10.1038/s43018-022-00448-0 PMC10150871 36357700

[B73] Toda M. Rabkin S. D. Kojima H. Martuza R. L. (1999). Herpes simplex virus as an *in situ* cancer vaccine for the induction of specific anti-tumor immunity. Hum. Gene Ther. 10 (3), 385–393. doi: 10.1089/10430349950018832 10048391

[B88] Todo T. Ino Y. Ohtsu H. Shibahara J. Tanaka M. (2022a). A phase I/II study of triple-mutated oncolytic herpes virus G47Δ in patients with progressive glioblastoma. Nat. Commun. 13 (1), 4119. doi: 10.1038/s41467-022-31262-y 35864115 PMC9304402

[B89] Todo T. Ito H. Ino Y. Ohtsu H. Ota Y. Shibahara J.. (2022b). Intratumoral oncolytic herpes virus G47Δ for residual or recurrent glioblastoma: a phase 2 trial. Nat. Med. 28, 1630–1639. doi: 10.1038/s41591-022-01897-x PMC9388376 35864254

[B79] Todo T. Martuza R. L. Rabkin S. D. Johnson P. A. (2001). Oncolytic herpes simplex virus vector with enhanced MHC class I presentation and tumor cell killing. Proc. Natl. Acad. Sci. U.S.A. 98 (11), 6396–6401. doi: 10.1073/pnas.101136398 11353831 PMC33479

[B74] Todo T. Rabkin S. D. Sundaresan P. Wu A. Meehan K. R. Herscowitz H. B.. (1999). Systemic antitumor immunity in experimental brain tumor therapy using a multimutated, replication-competent herpes simplex virus. Hum. Gene Ther. 10 (17), 2741–2755. doi: 10.1089/10430349950016483 10584921

[B153] Toll S. A. Tran H. N. Cotter J. Judkins A. R. Tamrazi B. Biegel J. A.. (2019). Sustained response of three pediatric BRAF(V600E) mutated high-grade gliomas to combined BRAF and MEK inhibitor therapy. Oncotarget 10 (4), 551–557. doi: 10.18632/oncotarget.26560 30728904 PMC6355184

[B137] Tomita Y. Kurozumi K. Yoo J. Y. Fujii K. Ichikawa T. Matsumoto Y.. (2019). Oncolytic herpes virus armed with vasculostatin in combination with bevacizumab abrogates glioma invasion *via* the CCN1 and AKT signaling pathways. Mol. Cancer Ther. 18 (8), 1418–1429. doi: 10.1158/1535-7163.MCT-18-0799 31092561

[B108] Uchida H. Marzulli M. Nakano K. Goins W. F. Chan J. Hong C. S.. (2013). Effective treatment of an orthotopic xenograft model of human glioblastoma using an EGFR-retargeted oncolytic herpes simplex virus. Mol. Ther. 21 (3), 561–569. doi: 10.1038/mt.2012.211 23070115 PMC3589172

[B38] Uneda A. Kurozumi K. Fujimura A. Fujii K. Ishida J. Shimazu Y.. (2021). Differentiated glioblastoma cells accelerate tumor progression by shaping the tumor microenvironment *via* CCN1-mediated macrophage infiltration. Acta Neuropathol. Commun. 9 (1), 29. doi: 10.1186/s40478-021-01124-7 33618763 PMC7898455

[B35] Wakimoto H. Kesari S. Farrell C. J. Curry W. T. Jr. Zaupa C. Aghi M.. (2009). Human glioblastoma-derived cancer stem cells: establishment of invasive glioma models and treatment with oncolytic herpes simplex virus vectors. Cancer Res. 69 (8), 3472–3481. doi: 10.1158/0008-5472.CAN-08-3886 19351838 PMC2785462

[B18] Wakimoto H. Mohapatra G. Kanai R. Curry W. T. Jr. Yip S. Nitta M.. (2012). Maintenance of primary tumor phenotype and genotype in glioblastoma stem cells. Neuro Oncol. 14 (2), 132–144. doi: 10.1093/neuonc/nor195 22067563 PMC3266381

[B40] Wang R. Chadalavada K. Wilshire J. Kowalik U. Hovinga K. E. Geber A.. (2010). Glioblastoma stem-like cells give rise to tumour endothelium. Nature 468 (7325), 829–833. doi: 10.1038/nature09624 21102433

[B70] Wang X. Li Y. Liu S. Yu X. Li L. Shi C.. (2014). Direct activation of RIP3/MLKL-dependent necrosis by herpes simplex virus 1 (HSV-1) protein ICP6 triggers host antiviral defense. Proc. Natl. Acad. Sci. U.S.A. 111 (43), 15438–15443. doi: 10.1073/pnas.1412767111 25316792 PMC4217423

[B37] Wang X. Prager B. C. Wu Q. Kim L. J. Y. Gimple R. C. Shi Y.. (2018). Reciprocal signaling between glioblastoma stem cells and differentiated tumor cells promotes malignant progression. Cell Stem Cell. 22 (4), 514–28.e5. doi: 10.1016/j.stem.2018.03.011 29625067 PMC5947947

[B44] Wouters R. Bevers S. Riva M. De Smet F. Coosemans A. (2020). Immunocompetent mouse models in the search for effective immunotherapy in glioblastoma. Cancers (Basel) 13 (1). doi: 10.3390/cancers13010019 PMC7793150 33374542

[B99] Xie R. Kessler T. Grosch J. Hai L. Venkataramani V. Huang L.. (2021). Tumor cell network integration in glioma represents a stemness feature. Neuro Oncol. 23 (5), 757–769. doi: 10.1093/neuonc/noaa275 33320195 PMC8099480

[B50] Xie X. P. Laks D. R. Sun D. Ganbold M. Wang Z. Pedraza A. M.. (2022). Quiescent human glioblastoma cancer stem cells drive tumor initiation, expansion, and recurrence following chemotherapy. Dev. Cell. 57 (1), 32–46.e8. doi: 10.1016/j.devcel.2021.12.007 35016005 PMC8820651

[B139] Xu B. Ma R. Russell L. Yoo J. Y. Han J. Cui H.. (2018). An oncolytic herpesvirus expressing e-cadherin improves survival in mouse models of glioblastoma. Nat. Biotechnol. 37, 45–54. doi: 10.1038/nbt.4302 PMC6535376 30475349

[B138] Xu B. Tian L. Chen J. Wang J. Ma R. Dong W.. (2021). An oncolytic virus expressing a full-length antibody enhances antitumor innate immune response to glioblastoma. Nat. Commun. 12 (1), 5908. doi: 10.1038/s41467-021-26003-6 34625564 PMC8501058

[B12] Yabo Y. A. Niclou S. P. Golebiewska A. (2022). Cancer cell heterogeneity and plasticity: a paradigm shift in glioblastoma. Neuro Oncol. 24 (5), 669–682. doi: 10.1093/neuonc/noab269 34932099 PMC9071273

[B154] Yoo J. Y. Swanner J. Otani Y. Nair M. Park F. Banasavadi-Siddegowda Y.. (2019). Oncolytic HSV therapy increases trametinib access to brain tumors and sensitizes them in vivo. Neuro Oncol. 21 (9), 1131–1140. doi: 10.1093/neuonc/noz079 31063549 PMC7571492

[B123] Zhang W. Fulci G. Wakimoto H. Cheema T. A. Buhrman J. S. Jeyaretna D. S.. (2013). Combination of oncolytic herpes simplex viruses armed with angiostatin and IL-12 enhances antitumor efficacy in human glioblastoma models. Neoplasia 15 (6), 591–599. doi: 10.1593/neo.13158 23730207 PMC3664991

[B136] Zhang G. Jin G. Nie X. Mi R. Zhu G. Jia W.. (2014). Enhanced antitumor efficacy of an oncolytic herpes simplex virus expressing an endostatin-angiostatin fusion gene in human glioblastoma stem cell xenografts. PloS One 9 (4), e95872. doi: 10.1371/journal.pone.0095872 24755877 PMC3995956

[B10] Zhang S. Rabkin S. D. (2021). The discovery and development of oncolytic viruses: are they the future of cancer immunotherapy? Expert Opin. Drug Discov. 16 (4), 391–410. doi: 10.1080/17460441.2021.1850689 33232188 PMC7969427

